# Allelic Variation of the Capsule Promoter Diversifies Encapsulation and Virulence In *Streptococcus pneumoniae*

**DOI:** 10.1038/srep30176

**Published:** 2016-07-28

**Authors:** Zhensong Wen, Yanni Liu, Fen Qu, Jing-Ren Zhang

**Affiliations:** 1 Center for Infectious Disease Research, School of Medicine, Tsinghua University, Beijing 100084, China; 2 The Center of Clinical Diagnosis, 302 Hospital of PLA, Beijing, China; 3 Collaborative Innovation Center for Biotherapy, State Key Laboratory of Biotherapy and Center, West China Hospital, West China Medical School, Sichuan University, Chengdu, China

## Abstract

The polysaccharide capsule is the major virulence factor of *Streptococcus pneumoniae* (pneumococcus), a major human pathogen. The sequences in the promoter and coding regions of the capsule gene locus undergo extensive variations through the natural transformation-mediated horizontal gene transfer. The sequence variations in the coding region have led to at least 97 capsular serotypes. However, it remains unclear whether the sequence polymorphisms in the promoter region have any biological significance. In this study, we determined the sequences of the *cps* promoter region from 225 invasive pneumococcal isolates, and identified modular composition and remarkable inter-strain sequence variations in this region. The strain-to strain variations in the *cps* promoter are characterized by diversity in sequence and size, mosaic combinations of nucleotide polymorphisms and sequence modules, selective preservation of the sequence combinations, and promiscuous assortments of the sequences between the promoter and coding regions. Isogenic pneumococci carrying allelic variants of the *cps* promoter displayed significant differences in the transcription of the capsule genes, capsule production, adhesion to host epithelial cells, anti-phagocytosis and virulence in mouse bacteremia model. This study has thus indicated that the sequence polymorphisms in the *cps* promoter represent a novel mechanism for fine-tuning the level of encapsulation and virulence among *S. pneumoniae* strains.

*Streptococcus pneumoniae* (pneumococcus) is a Gram-positive bacterium that naturally resides in the human nasopharynx, but it causes a wide spectrum of bacterial infections including community-acquired pneumonia, otitis media, bacteremia, and meningitis^1^. Despite extensive use of antimicrobials and vaccines*, S. pneumoniae* remains as a major cause of bacterial infections worldwide, leading to approximately 1 million of death annually^2^,^3^. The polysaccharide capsule is the major virulence factor of *S. pneumoniae*, which enables the pneumococci to evade phagocytic killing by host immune system due to its negative charge and other biochemical properties^4^,^5^,^6^. However, the capsular polysaccharide (CPS) is immunogenic, and host immunity targeting the CPS is protective against the infection by the pneumococcus carrying the same CPS^7^. As a result of host immune selection, *S. pneumoniae* has evolved to produce at least 97 chemically and antigenically distinct types of the capsule (or serotypes) by frequent replacement of the CPS biosynthesis genes though acquisition of foreign DNA by natural genetic transformation^8^. Each type of CPS is synthesized and exported by a unique capsule gene (for type-37) or a set of the capsule genes (for all other types)^9^.

The capsule genes of all CPS types are clustered in the same locus of the genome between the *dexB* and *aliA* genes with the exception of the type-37 synthase gene (*tts*)^5^,^9^. The *tts* gene is located elsewhere on the chromosome due to degeneration of the original capsule genes in the *dexB*-*aliA* locus^10^. There are extensive inter-strain sequence variations in both the promoter and coding regions of the capsule locus as exemplified by the diversity in the locus size of different strains/types, which ranges from approximately 2.2 kilobases (kb) (type-37) to 30 kb (type-38) with an average of 21 kb^9^. The *cps* locus of the type-2 strain D39, the most extensively studied virulent *S. pneumoniae* strain, consists of 17 CPS-biosynthesis associated genes (20.316 kb in size)^11^. The sequence diversity in the coding region of the *cps* locus is responsible for the enormous diversity of the CPSs in chemical structure and antigenic property (a basis for pneumococcal serotyping).

Extensive sequence variations have also been described in the promoter region of the *cps* locus, which is defined as the intergenic sequence between *dexB* and *cpsA* (the first gene in the *cps* locus)^12^,^13^. Despite of its importance in the capsule production, the promoter region of *S. pneumoniae* is poorly characterized. As described in type-2 strain D39^12^, the typical promoter region consists of four basic modules upstream of the *cpsA* gene, the first gene in the capsule locus: insertional element (IE), repeat unit of pneumococcus (RUP), spacing sequence (SS), and a core promoter sequence that was previously defined in type-4 strain TIGR4 by Shainheit *et al*.^14^. The core promoter contains the predicted −10/−35 promoter motifs and the transcriptional start site^14^. Our previous study showed that the 17-gene *cps* locus in strain D39 is transcribed as an operon from the *cps* promoter region of the *cps2A*; the full transcription of the *cps* genes requires not only the core promoter, but also the other upstream modules (e.g., IE, RUP, and SS)^12^.

Based on sequence variation in the *cps* promoter region, Moscoso *et al*. identified 26 sequence organizations (SOs) from the sequences of the *cps* promoter from 115 *S. pneumoniae* sequence entries; the majority of the SOs consists of one or more IEs, a RUP, and a conserved sequence (covering the core promoter and spacing sequence)^13^. However, it remains completely undefined whether the inter-strain allelic variations in the *cps* promoter region have any impact on pneumococcal encapsulation and virulence. In this study, we performed a comprehensive sequencing analysis of the *cps* promoter region in 225 invasive isolates of *S. pneumoniae*. Among the *cps* promoter variants of these isolates, our data revealed: 1) extensive variations in sequence and size, 2) mosaic combinations of nucleotide polymorphisms and sequence modules, 3) selective preservation of the sequence combinations, and 4) promiscuous assortments of the promoter sequences with those of the coding region. Functional analysis of the selected promoter sequences uncovered significant impact of the allelic variations in this region on the transcription of the *cps* genes, capsule production, adhesion to host epithelial cells, anti-phagocytosis and virulence in isogenic pneumococcal backgrounds. These data indicate that the allelic variations in the *cps* promoter serve as a novel mechanism for modulating the amount of pneumococcal capsule, pathogen-host interaction and virulence.

## Results

### The *cps* promoter of invasive pneumococcal isolates contains distinctive combinations of nucleotide polymorphisms and sequence modules

There are extensive strain-to-strain sequence variations in the *cps* promoter region of *S. pneumoniae*, or the intergenic region between the *dexB* and *cpsA* genes^12^,^13^. However, it remains completely unclear whether the promoter allelic variations are relevant to the biological behaviors of this pathogen, particularly to its encapsulation and virulence. This study attempted to evaluate potential pathobiological impact of the promoter polymorphisms in invasive pneumococci. Because many existing sequence entries of the *cps* promoter in the public databases did not provide the necessary background information of the source strains (e.g., serotype and tissue/geographic source), which was essential for our investigation of invasive pneumococci, we performed sequencing analysis of the *cps* promoter region from 225 invasive isolates of *S. pneumoniae*. These isolates represented at least 30 capsular serotypes (1, 4, 6A, 6B, 7F, 8, 9A, 9V, 10, 10A, 11, 12F, 14, 15A, 15B, 15C, 16F, 18C, 19A, 19F, 20, 22F, 23B, 23F, 29, 33, 33F, 34, and 35B), including the vast majority of the serotypes covered by the current pneumococcal vaccines^15^. Because the capsule genes of the type-3 strains are completely different from and not comparable with those of all the other types, our sequence analysis focused only on the non-type-3 isolates.

**Figure 1:**
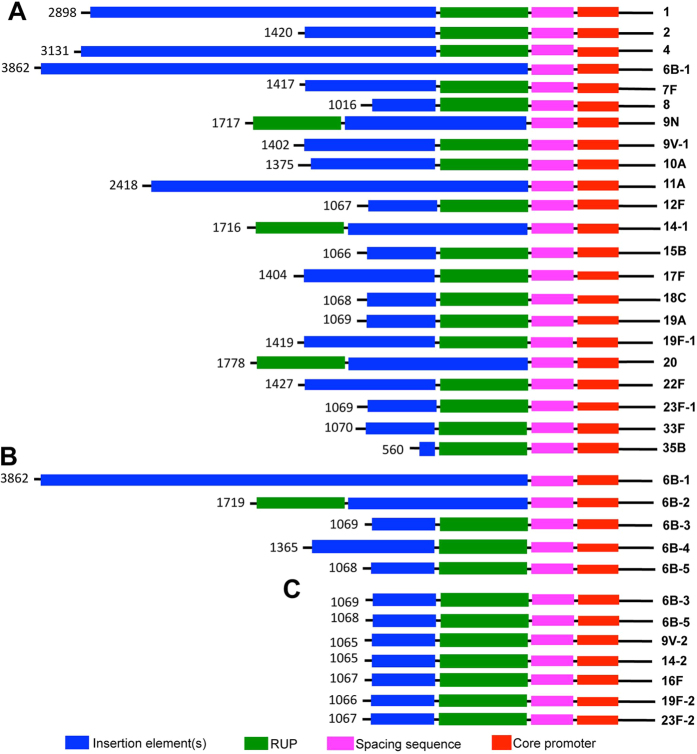
Diagrammatic illustration of sequence modules and their relative positions in the *cps* promoter region of invasive pneumococcal isolates. (**A**) Sequence diversity among the strains representing the 22 WZY-dependent IPD types. (**B**) Sequence diversity among the five type-6B promoter strains used in Figs 6 and 7. (**C**) Independence of the promoter sequence from the coding region (capsular type). Sequence modules are indicated with colored strips at the bottom. The size of each sequence (bp) and serotype of the source strain are indicated at the left and right sides, respectively. The numbers following the serotype icons indicated the different strains of the same serotypes.

**Figure 2:**
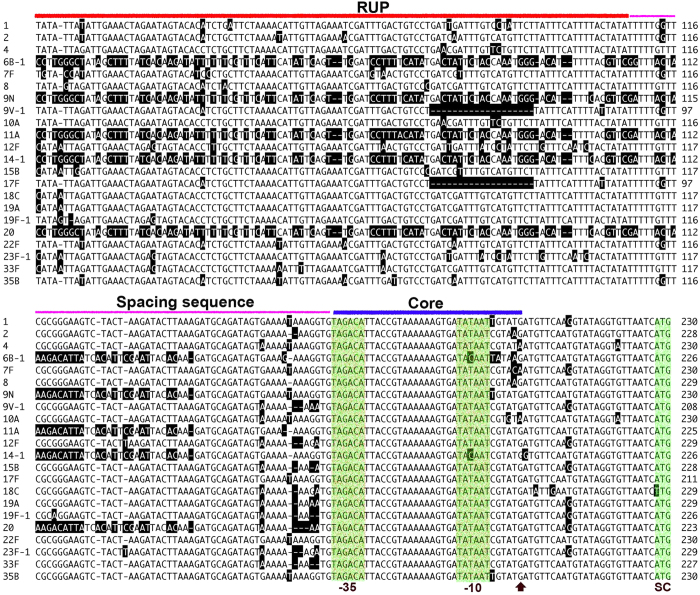
Alignment of the partial *cps* promoter sequences from the 22 WZY-dependent IPD types. The region covering the RUP, spacing sequence, and core promoter modules were aligned with the sequences from the strains illustrated in Fig. 1A by the Cluster W method^58^. The IE module is not fully presented due to their extensive sequence differences and space limit. Partial IE module is included for each of the strains lacking the RUP module immediately upstream of the SS module. The promoter motifs (−10 and −35), transcriptional start site identified in type-2 strain D39^12^, *cpsA* start codon (SC) were indicated at the both of the second panel.

These isolates displayed various levels of sequence variations in the *cps* promoter region, reflected by differences in the arrangement of sequence modules, molecular size, and nucleotide polymorphisms (Fig. 1 and Fig. S1). As represented in Fig. 1A, there were several unique arrangements of sequence modules (e.g., IE, RUP, SS, and core promoter) in this region. All of the sequences contained a relatively conserved segment in the 3′ ends (approximately 85 bp) although polymorphisms were still present virtually in all of the strains. This region consists of a partial SS module (~26 bp), the core promoter (~35 bp), and the ribosome binding site-containing 5′ untranslated segment (~24 bp) (Fig. 2). The upstream IE, RUP, and 5′ SS modules are highly variable in sequence and arrangement, which were responsible for the diversity in the size among the *cps* promoter variants. The RUP module was present in the vast majority of the invasive isolates, but its relative position was not a constant (Fig. 1). RUP was positioned between the IE and SS modules in the vast majority of the isolates (Fig. 1A, rows 1–3), but it was also found upstream of the IE module in certain isolates (Fig. 1A, 9N, 14–1, and 20). RUP was entirely absent from 20 out of the 225 invasive isolates (9%) tested in this work.

The *cps* promoter sequences of many isolates also exhibited remarkable differences in molecular size, particularly in the IE and RUP modules. IE represents the most variable segment in the *cps* promoter region, ranging from >200 bp to <3,000 bp, and is primarily responsible for the size differences among the isolates. The longest promoter sequence (3,862 bp) was identified in type-6B isolate TH2882 (Fig. 1A, 6B-1), which is identically present in the genome of strain 670-6B (accession CP002176). Although this sequence was highly divergent from the counterparts of many other strains due to its long IE and lack of the RUP module, it is structurally similar to that of type-11A strain TH4849 (Fig. 1A, 11A; 2,418 bp). The latter lacked a 5′ insertion element (1,441 bp) of strain TH2882, which was responsible for the size difference between the two sequences. The sequence from type-35B isolate TH2733 had the shortest sequence (560 bp in size) (Fig. 1A, type-35B). This sequence consisted of an extremely truncated IE module (192 bp), a 107-bp RUP, a 61-bp spacing sequence, and the core promoter.

Intriguingly, there is no apparent association between the capsule types and the modular/sequence features of the *cps* promoter. First, the *cps* promoter sequences from the strains within the same serotype could be as divergent as those from the strains with different capsular types. As exemplified in Fig. 1B and Fig. S2, the *cps* promoter sequences from the five type-6B strains remarkably differed in size and modular arrangement. For example, the sequence of strain TH2882 (Fig. 1B, 6B-1) was 2–3 times larger than those of the other 6B strains. Instead, this sequence is more similar to the *cps* promoter sequence of type-11A strain TH4849 (Fig. 1A, 11A). Along this line, unlike the other type-6B isolates, strain TH2787 (Fig. 1B, 6B-2) positioned its RUP module in front of the IE module, resembling the modular structure of the promoter sequences in the type-9N, -14, and -20 strains (Fig. 1A). Second, pneumococcal strains with different types of the capsule carried similar promoter sequences in the capsule locus. As exemplified in Fig. 1C and Fig. S3, the capsule locus in strains of types 6B (6B-3, 6B-5), 9V, 14, 16F, 19F, and 23F possessed highly homologous promoter sequences as reflected by their similarities in molecular size and modular arrangement. Promiscuous assortments of the promoter sequences with those of the coding region in these invasive isolates implicate that the promoter and coding regions of the *cps* locus are able to undergo independent assortments by horizontal gene transfer through natural genetic transformation.

Lastly, there are many minor polymorphisms throughout the four basic modules of the entire promoter among these isolates, which include nucleotide replacements, insertions, and deletions (Fig. 2, Figs S2 and S3). Interestingly, some of the sequence changes occurred in the predicted -10 promoter motif, although the vast majority of the isolates had a sequence of 5′-TATAAT-3′. However, these nucleotide polymorphisms in the *cps* promoter, as well as the arrangements of the sequence modules, are individually shared by many isolates although the combinations of these changes resulted in the mosaic pattern. These lines of evidence suggest that the allelic variations in the *cps* promoter have been maintained and shared by the pneumococcal strains as a function of selective pressure in humans. Taken together, our sequencing result has uncovered extensive variations in sequence and modular structure of the *cps* promoter. The sequence and modular features of the *cps* promoter are not associated with the boundary of the capsular types (the coding region in the capsule locus), suggesting that the promoter and coding regions of the *cps* locus are able to undergo independent assortments by horizontal gene transfer through natural genetic transformation.

### Strain-to-strain sequence variations in the *cps* promoter of the major invasive pneumococcal disease (IPD) types alter the transcription of the capsule genes

Our recent study has shown that the full transcription of the *cps* operon and capsule production depend on the four sequence modules in the *cps* promoter region^12^. Based on the extensive sequence variations in these promoter modules, particularly in the IE and RUP (Fig. 1), we hypothesized that the inter-strain sequence polymorphisms in the *cps* promoter affect the transcription of the capsule gene operon. To test this possibility, we initially determined the transcriptional activity of the *cps* promoter sequences from the strains representing the 22 major IPD serotypes in the pneumococcal vaccines using a luciferase-based transcriptional reporter system. The entire sequence between the *dexB* stop codon and *cpsA* start codon was amplified by PCR from each target strain using the same primer pair (except for the sequence in the type-3 strain), fused to the upstream of the luciferase gene in the pIB166 shuttle plasmid, and transformed into D39, a type-2 virulent Avery strain^16^. The type-3 *cps* promoter was amplified with the primer sequences in the upstream region of the *ugd* (*cps3D*) gene^9^. The relevant information for the reporter construction is provided in Table S2.

**Figure 3:**
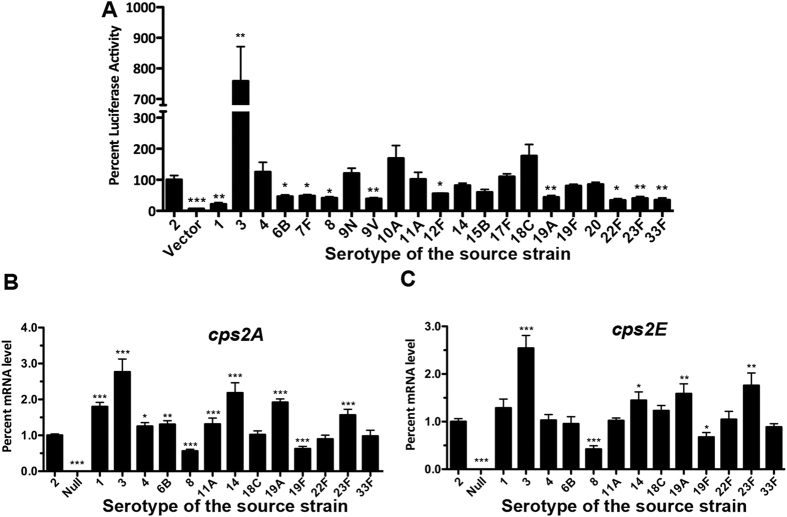
Transcriptional activity from the promoter sequence for each of the 22 major IPD serotypes. (**A**) Assessment of promoter activity by a luciferase reporter. Each promoter sequence was fused to the 5′ end of a promoterless luciferase gene in the pIB166 shuttle plasmid, and transformed in strain D39. The promoterless plasmid (vector) was used as a negative control. Transcriptional activity of each *cps* promoter sequence was quantified by measuring luminescence level of the mid-log culture inoculated with the pneumococci carrying the appropriate reporter construct. Each reporter was identified with the serotype of its source strain. The sequence of the first strain was used for each of the serotypes with multiple strains listed in Fig. 1. The values represent the percent luciferase activities of the reporters relative to the luciferase activity readout of the D39 (first bar) promoter construct. (**B**) The qRT-PCR readouts of the *cps2A* transcripts from the promoter replacement D39 derivatives of 13 IPD serotypes. The promoterless derivative TH4525 described in our previous study was used as a negative control. The mRNA levels were presented as relative values to that of D39s. (**C**) Same as panel B, except that *cps2E* was tested. The sequence of the first strain was used in both the luciferase and promoter replacement experiments for each of the serotypes with multiple strains listed in Fig. 1. The values were the means ± standard errors of the results from triplicate samples as compared with those of the type 2. *p < 0.05, **<0.01 and ***<0.001.

When compared with the luciferase activity of the type-2 *cps* promoter (as a reference), the *cps* reporter constructs showed variable levels of transcriptional activity (Fig. 3A and Table S4). The type-3 construct showed the highest transcription activity among all of the 22 *cps* reporters with a 6.58-fold increase above the reference level. Although the margins of the variations were relatively small, 10 additional reporters displayed relatively lower levels of luciferase activity, such as those of types 1 (21.0%), 6B (46.0%), 7F (48.3%), 8 (41.3%), 9V (38.6%), 12F (55.3%), 19A (43.5%), 22F (33.9%), 23F (40.4%), and 33F (34.2%). The remaining 10 constructs showed a level of luciferase activity similar to that of the D39 (e.g., 4, 9N, 10A, 11A, 14, 15B, 17F, 18C, 19F, and 20). The reporter experiment suggested that sequence diversity in the *cps* promoter among different strains/types affects the transcription of the *cps* operon.

We subsequently sought to verify the transcriptional impact of the *cps* promoter sequence polymorphisms with a series of isogenic promoter replacement derivatives of strain D39, with a focus on the promoter sequences displaying significant differences in the reporter test (Fig. 3A). The replacement strains were constructed by individually replacing the *dexB-cps2A* region of D39 with the corresponding region from the target strains through counter selection. The transcriptional activities of the “transgenic” promoters were assessed by determining the mRNA levels of *cps2A* and *cps2E* in the resulting D39 derivatives by quantitative real-time PCR (qRT-PCR). The *cps2A* and *cps2E* were previously used as the transcriptional readouts of the *cps* operon^12^,^14^. The *cps2A* primers revealed significant differences between the type-2 and all of the 12 *cps* promoter sequences except for those of types 18C, 22F, and 33F (Fig. 3B). The *cps2E* amplification identified much fewer significant hits with significant differences in 6 of the 12 *cps* promoter sequences (types 3, 8, 14, 19A, 19F, and 23F) (Fig. 3C). Consistent with the luciferase reporter data, the strain containing the type-3 promoter displayed the highest transcription levels of both *cps2A* (by 1.76-fold) and *cps2E* (by 1.54-fold). Although the overall transcriptional patterns of the *cps2A* and *cps2E* were similar for most of the *cps* promoter replacement strains, discrepancy was observed with the *cps2A* and *cps2E* results with the strains containing the *csp* promoter sequences of types 1, 4, 6B, and 11A. As compared with the parent strain, these strains showed significantly higher levels of *cps2A* (Fig. 3B) but not *cps2E* (Fig. 3C). Given both *cps2A* and *cps2E* are co-transcribed^12^, this discrepancy may reflect uneven degradation of different regions in the *cps* mRNA. As exemplified by the *cps* promoter sequence of types 1, 4, 6B, 11A, 14, 19A, 19F, 22F, 23F, and 33F, we also observed apparent inconsistency between the results of the luciferase reporter and qRT-PCR experiments, which could be caused by relative stability of the luciferase reporter as compared with the fast degradation of the *cps* mRNA as indicated in our previous Northern blotting analysis^12^. Taken together, the transcriptional reporter and qRT-PCR experiments showed that certain inter-strain/type sequence variations in the *cps* promoter lead to significant changes in the transcription of the capsule locus.

### Inter-type sequence polymorphisms in the *cps* promoter of the major IPD types alter the level of the pneumococcal encapsulation

**Figure 4:**
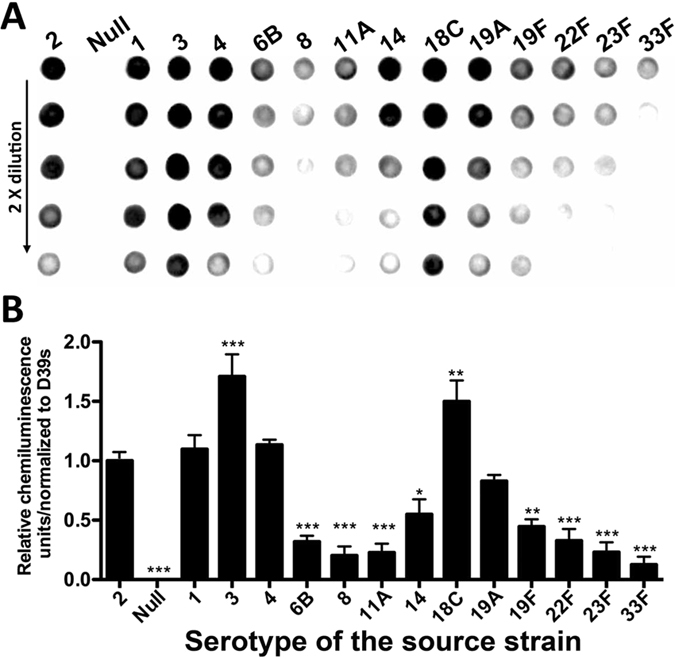
Detection of CPS production in D39s and its *cps* promoter replacement derivatives. The CPS of the pneumococci was assessed by immunoblotting using an antiserum specific for the type-2 CPS signal (**A**). The signal intensity on each spot was digitized with ImageJ and normalized to the corresponding value of D39s (**B**). The value of each bar was the mean ± standard error of the result from all of the detectable spots (>3) for each sample after considering dilution factors and being compared with the type-2 parental strain. *p < 0.05, **<0.01 and ***< 0.001.

Significant differences in the transcriptional capacity among the *cps* promoter sequences promoted us to determine whether these inter-strain/type sequence variations affect pneumococcal capsule production. The CPS level of the promoter replacement strains was determined by immunoblotting with an antibody against type-2 pneumococcal CPS. This experiment showed dramatic differences among most of the promoter replacement strains in the level of the capsule production (Fig. 4A). Consistent with its stronger transcriptional capacity (Fig. 3), the type-3 promoter produced the highest level of CPS, which was 71% higher than that of strain D39s (Fig. 4B). The 18C promoter also yielded 50% more CPS than the parent strain although no significant difference in the transcriptional capacity was detected between the two strains (Fig. 3). In contrast, significantly lower levels of the CPS were detected in the promoter replacement strains of types 6B (32%), 8 (20%), 11A (23%), 14 (55%), 19F (45%), 22F (33%), 23F (23%), and 33F (13%) (Fig. 4B). Importantly, each of these promoter sequences yielding the lower CPS production is also carried by many other strains from multiple capsular serotypes. For example, the sequence of the type-33F *cps* promoter used in this work shares the same modular structure and highly similar sequence with those of the strains representing at least 23 different capsular types (e.g., 6B, 7B, 10B, 10F, 12B, 12F, 14, 15A, 18B, 18C, 19F, 19B, 19F, 23A, 23F, 27, 29, 44, 46, 28F, 33A, 35A, and 35B).

The reduced CPS production in these reporter strains could be explained by the relatively lower transcription capacity of the corresponding *cps* promoters in the luciferase reporter and/or qRT-PCR experiments. Overall, the immunoblotting results were more consistent with the data obtained by the luciferase reporters (Fig. 3A), suggesting this is a more robust predictor of the capsule production. Together with the transcriptional detection results, the immunoblotting data indicated that the sequence polymorphisms in the *cps* promoter are capable of generating remarkable diversity in the transcription of the capsule genes and pneumococcal capsule production.

### Inter-type sequence polymorphisms in the *cps* promoter of the major IPD types modulate pneumococcal pathogenic properties

**Figure 5:**
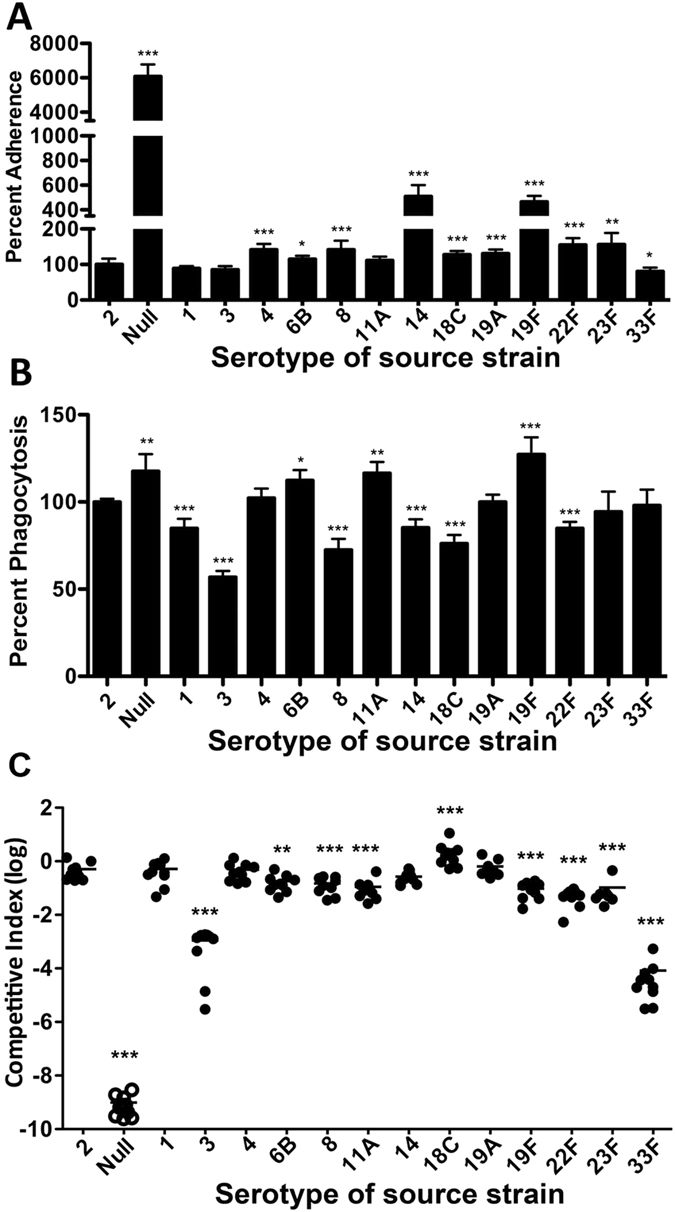
Epithelial adhesion, anti-phagocytosis and virulence of D39s and its *cps* promoter replacement derivatives. (**A**) Pneumococcal adhesion to human alveolar epithelial A549 cells. D39s and its promoter replacement derivatives were incubated with the confluent monolayers of A549 cells for 1 h before the monolayers were lysed to enumerate the number of viable pneumococci on TSA blood plates. The adhesion levels of the promoter replacement strain are displayed as the percentage values relative to the value in D39s. (**B**) Phagocytosis of the *cps* promoter derivatives by RAW264.7 murine macrophages. The RAW264.7 cell monolayers were infected with the pneumococci, treated with antibiotics, and lysed to quantify intracellular (phagocytosed) bacteria as in A. The phagocytosis level of each promoter replacement strain was shown as the percentage value relative to the value of the parent strain D39s. (**C**) The virulence levels of the promoter replacement strains. CD1 mice were intraperitoneally infected with a mixture of D39 and one of the promoter replacement strains in a 1:1 ratio. The pneumococci in the bloodstream of the mice were enumerated 21 h post infection as in A. Each filled circle represents a relative bacteremia level or competitive index (CI) value of a single mouse. The open circles indicate the mice, from which no unencapsulated pneumococci were recovered. The horizontal bar indicated the mean in each group of mice. The parental strain (type-2) was used as a reference. *p < 0.05, **<0.01 and ***< 0.001.

The pneumococcal capsule hinders bacterial attachment to mammalian cells^17^. We reasoned that pneumococcal adhesion to epithelial cells may be affected by variations in the level of encapsulation. We first compared the levels of epithelial adhesion among the isogenic promoter replacement strains. As a control, the *cps* promoter-null strain showed a 60-fold more adhesion than the encapsulated parent strain (D39s) (Fig. 5A), confirming the blocking effect of the capsule on pneumococcal binding to host cells likely through masking the adhesive molecules on the cell surface. Consistent with the relatively thicker capsule (Fig. 4), the strains carrying the *cps* promoters of types 14 (by 4-fold) and 19F (by 3.6-fold) showed significantly higher levels of adhesion than the parent strain. To less extents, significant increase in adhesion was also observed with the strains carrying the promoters of types 4, 6B, 8, 19A, 22F, and 23F. Surprisingly, the strain with the lowest CPS production (33F) did not show the anticipated (highest) level of adhesion. Along the same line, the type-3 and -18C promoter strains did not show expected reduction in adhesion based on their CPS production levels. Nevertheless, this result demonstrated that certain inter-type sequence variations in the *cps* promoter lead to significant phenotypic impact on pneumococcal adhesion.

It is believed that inhibiting pneumococcal attachment to host phagocytes is a major mode of action for the capsule^5^. We thus determined potential impact of promoter sequence variations on pneumococcal phagocytosis in the RAW 264.7 murine macrophage. As a control, the encapsulated mutant showed substantial increase in phagocytosis (Fig. 5B, null), although the extent of the increase (18%) in phagocytosis was much less than in epithelial adhesion (Fig. 5A). In agreement with the capsule production (Fig. 4), the strains with the type-3 and -18C promoters showed relatively lower levels of phagocytosis, whereas those with the *cps* promoters of types 6B, 11A, and 19F displayed significantly enhanced uptake by the macrophages. The phagocytosis in the types 8, 14, and 22F strains could not be explained simply by their CPS levels (Fig. 4, Table S4).

We next assessed the relative virulence of these strains by intraperitoneal co-infection of CD1 mice with a 1:1 mixture of D39 and one of the promoter replacement strains. The level of viable bacteria in the bloodstream of mice (bacteremia) 21 h postinfection was used as a marker for pneumococcal virulence. The wild type D39 (streptomycin sensitive) was used as a reference strain in the co-infection experiments for the purpose of differentiating the parent strain from its streptomycin-resistant derivative of D39s. The streptomycin-resistant derivative of D39 (D39s) was slightly attenuated in this mouse model because the ratio between the mutant and wild type bacteria recovered from each co-infected mouse was approximately 0.5:1 (~50% attenuation).

All of the promoter replacement strains displayed significant variations in virulence, except for those carrying the promoters of types 1, 4, 14, and 19A (Fig. 5C). In agreement with the CPS immunoblotting result, seven of the eight strains with reduced CPS (except for type-14) displayed significant attenuation in the bacteremia model (types 6B, 8, 11A, 19F, 22F, 23F, and 33F). Among these, the type-33F reporter exhibited the most severe attenuation (by 6,249-fold), which was also the poorest CPS producer (Fig. 4). The remaining six strains showed much less but significant attenuation (types 23F by 10.5-fold, 22F by 9-fold, 19F by 4-fold, 11A by 3.6-fold, 8 by 2-fold, and 6B by 1.3-fold). Consistent with the 50% increase in CPS production over the parent strain, the strain carrying the type-18C promoter showed a 4.3-fold enhancement in bacteremia level. This result suggested that pneumococcal growth in the bloodstream is proportionally enhanced by the CPS level. Contradictory to this notion, the type-3 reporter strain was dramatically attenuated by 451-fold although it synthesized 71% more CPS over the parent strain (Fig. 4), indicating that excessive production of the capsule is detrimental to pneumococcal fitness during the systemic infection. Consistent with our previous study^12^, D39s (the parent strain) showed a minor attenuation in virulence as compared with the wild type D39 (1-fold reduction), which was factored in our calculation of the final CI values. Finally, no viable bacteria were recovered from any of the 12 mice infected with the *cps* promoter-null strain TH4525 (Fig. 5C), confirming the essentiality of the capsule in pneumococcal virulence. These data demonstrated that many allelic variants of the *cps* promoter in the major IPD serotypes are able to generate profound phenotypic diversity in the capacity of pneumococcal virulence.

### Inter-strain polymorphisms in the type-6B *cps* promoter alter the levels of pneumococcal encapsulation, epithelial adhesion, anti-phagocytosis and virulence in the D39 background

As exemplified in Fig. 1B, our earlier sequencing analysis revealed extensive inter-strain sequence variations in the *cps* promoter within many pneumococcal IPD serotypes, including type-6B. Based on the striking phenotypic impact of sequence variations in the *cps* promoter among the strains representing the major IPD serotypes, we further determined whether this functional influence operates among the *cps* promoter sequences within type-6B, because type-6B is a major international IPD serotype in both children and adults^1^, and was abundantly represented in the IPD strains that were sequenced in this study.

**Figure 6:**
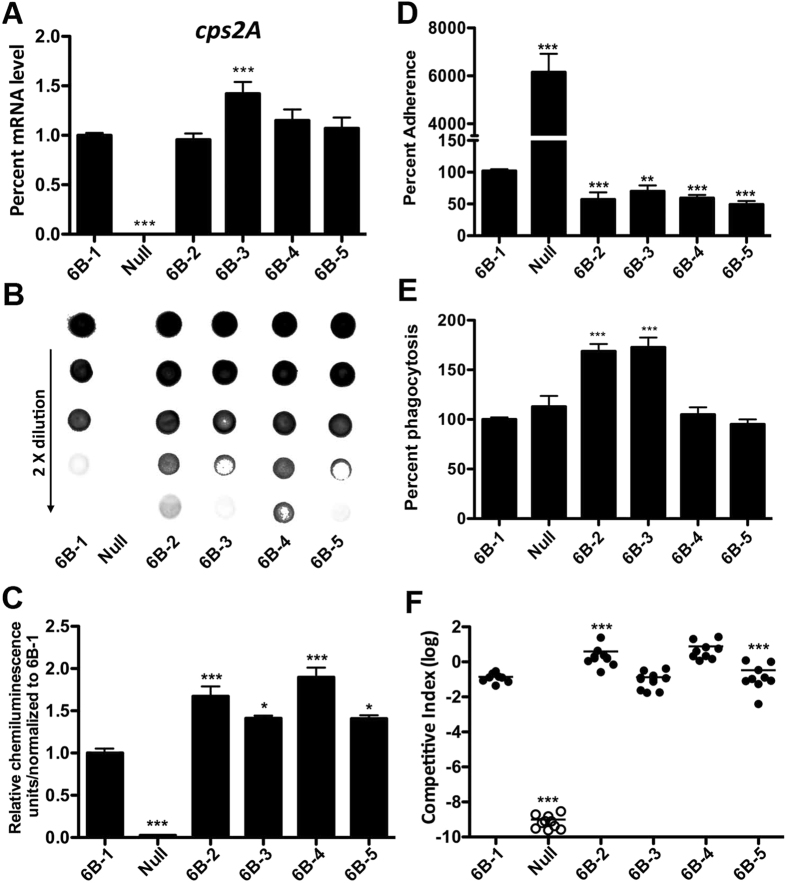
Phenotypic impact of the sequence variation in the type-6B *cps* promoter in the D39 background. The *cps* promoter sequence from each of the five type-6B invasive isolates were amplified and used to replace the *cps* promoter of strain D39s. The resultant strains (from 6B-1 to 6B-5) were tested for their levels of transcription in *cps2A* (**A**), capsule production (**B,C**), epithelial adhesion (**D**), phagocytosis (**E**), and virulence (**F**). The values in each set of the experiments were normalized to those of strain 6B-1, and presented as in Figs 3, 4, 5. *p < 0.05, **<0.01 and ***< 0.001.

We initially generated reporter derivatives of strain D39s for the five *cps* promoter sequences of type-6B (represented in Fig. 1B, Fig. S2). These sequences were selected because they represented the diversity of the *cps* promoter, in terms of nucleotide sequence, size, and modular arrangement. The sequences between the *dexB* and *cps6BA* of type-6B strains were amplified from genomic DNA samples of five original 6B strains (Table 1), and used to generate the unmarked promoter replacement derivatives in D39s by counter selection as described in Fig. 3. The *cps* transcriptional activity and capsule production of each promoter replacement strain were assessed by qRT-PCR and immunoblotting, respectively. The strain carrying the longest type-6B *cps* promoter (Figs. 1B, 6B-1) was used as a reference for comparative analysis.

The qRT-PCR analysis of the *cps2A* (Fig. 6A) and *cps2E* (Fig. S4A) transcripts showed a comparable transcription activity for four of the five type-6B *cps* promoter sequences (6B-1, 6B-2, 6B-4, and 6B-5). The strain carrying the 6B-3 promoter sequence had significantly higher levels in the *cps2A* (by 41%) and *cps2E* (by 55%) transcription. However, the immunoblotting analysis revealed that this strain along with 6B-3 and 6B-5 produced substantially lower levels of CPS than strains 6B-2 and 6B-4; strain 6B-1 produced the least amount of CPS (Fig. 6B). The slightly more transcription of the *cps* genes in strain 6B-3 did not result in more CPS. The results obtained with the five type-6B *cps* promoter sequences thus confirmed the earlier immunoblotting analysis (Fig. 4) that inter-strain sequence variations in the *cps* promoter lead to significant functional impact on the capsule production of *S. pneumoniae*.

We further determined the effect of the type-6B *cps* promoter polymorphisms on pneumococcal adhesion, phagocytosis and virulence. Consistent with the immunoblotting results, strain 6B-1 with the lowest CPS showed a small but significantly higher level of epithelial adhesion to A549 cells than the rest of the five strains (by 30–50%) (Fig. 6D), verifying that the pneumococci with a thinner capsule are more capable of attaching to host cells. To our surprise, the 6B-2 and 6B-3 strains showed significantly enhanced phagocytosis in the RAW 264.7 murine macrophages (Fig. 6E and Table S4), suggesting that this phagocytosis model is not suitable for assessing capsular impact on pneumococcal phagocytosis. In full concordance with their encapsulation levels, the type-6B *cps* promoter replacement strains displayed two different levels of virulence in the mouse bacteremia model (Fig. 6F). The two strains producing thicker capsules (6B-2 and 6B-4) were much more virulent than the three counterparts with relatively lower levels of CPS (6B-1, 6B-3, and 6B-5). Specifically, the mice infected with strains 6B-2 and 6B-4 had 27.8- and 55.2-fold more pneumococci in the bloodstream than the 6B-1-infected animals, respectively. These results demonstrated that the sequence polymorphisms in the *cps* promoter among the strains of the same serotype significantly affect pneumococcal adhesion and virulence.

### Inter-strain polymorphisms in the type-6B *cps* promoter diversify pneumococcal encapsulation, epithelial adhesion, anti-phagocytosis and virulence in the ST858 (type-6B) background

**Figure 7:**
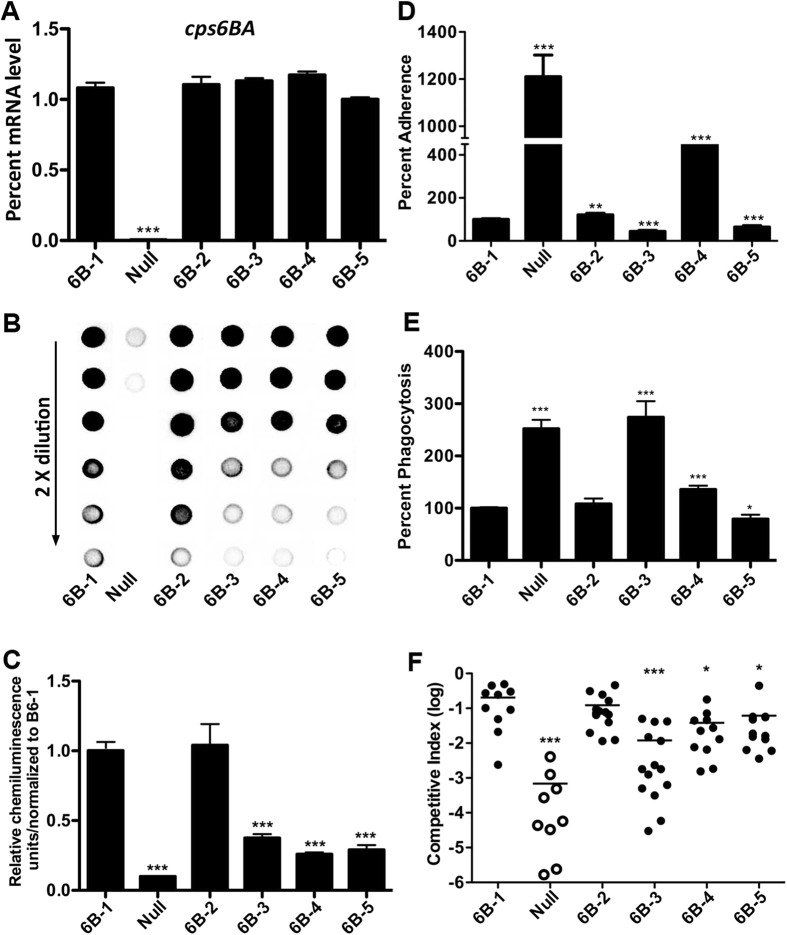
Phenotypic impact of the sequence variation in the type-6B cps promoter in the ST858 (type-6B) background. The *cps* promoter replacement derivatives were constructed in strain ST858 with the promoter sequences of 6B-1-5, and tested for *cps* transcription (**A**), capsule production (**B,C**), epithelial adhesion (**D**), phagocytosis (**E**), and virulence (**F**) as in Fig. 6. *p < 0.05, **<0.01 and ***< 0.001.

To rule out potentially strain-specific effect in the experiments conducted in the D39 background (Fig. 6), we placed the same sets of the type-6B *cps* promoters in the *dexB-cps6BA* intergenic region of strain ST858, a virulent type-6B blood isolate and the natural carrier of the 6B-5 *cps* promoter (Fig. 1B). The *cps* promoter replacement derivatives were generated in strain ST858 as in D39. The resulting strains were used to characterize their activities in the *cps* gene transcription, type-6B capsule production, epithelial adhesion, anti-phagocytosis and virulence as described above. The strain carrying the 6B-1 *cps* promoter was used as a reference for comparison. The qRT-PCR analysis revealed similar levels of the *cps6BA* and *cps6BE* transcripts among the five promoter replacement strains (Fig. 7A and Fig. S4B). This result was comparable with what was observed with the D39 derivatives of the same *cps* promoter sequences, with the exception of the 6B-3 construct (Fig. 6A and Fig. S4A). However, the immunoblotting test showed that the 6B-1 and 6B-2 strains were more abundantly encapsulated than the other three promoter replacement strains (6B-3, 6B-4, and 6B-5). The profile of epithelial adhesion was similar to that of the CPS production, except for the 6B-4 strain (Fig. 7D). The 6B-4 promoter yielded 3.7-fold more adhesion than the reference strain. In agreement with epithelial adhesion, the 6B-4 and 6B-5 strains displayed substantially changed phagocytosis in the RAW 264.7 murine macrophage (Fig. 7E and Table S4), but the phagocytosis result of strain 6B-3 was opposite to the epithelial adhesion data (Fig. 7D–E), which could be caused by potential bacterial killing in the RAW 264.7 cells during the assay. Because the 6B-4 promoter resulted in comparable adhesion with the other four promoters in the D39 background (Fig. 6D), this result suggested that this sequence may be involved in background-specific pneumococcal interactions with host cells.

We finally assessed the functional impact of the type-6B *cps* promoter polymorphisms on the virulence of strain ST858 in the mouse bacteremia model. The streptomycin-resistant derivative of ST858 (ST858s) was slightly attenuated in this mouse model because the ratio between the mutant and wild type bacteria recovered from each co-infected mouse was approximately 0.5:1 (~50% attenuation). As in the D39 background (Fig. 6F), the same type-6B promoter sequences yielded distinct levels of bacteremia in ST858 (Fig. 7F). While the 6B-1 and 6B-2 strains showed a similar levels of bacteremia, significantly lower levels of bacteremia were observed with the counterparts carrying the other three promoters. As compared with strain 6B-1, 6B-3 (by 16-fold), 6B-4 (by 4.3-fold), and 6B-5 (by 2.3-fold) were substantially attenuated in the capacity of growth in the bloodstream. As a control, the promoterless mutant of ST858 was totally avirulent (without any detectable pneumococci from the blood of the infected mice). The virulence level of each ST858 derivative was in concordance with the amount of the CPS that was detected in the *in vitro* cultured pneumococci. These results demonstrated that the promoter sequence polymorphisms in the *cps* locus of type-6B strains are capable of diversifying the amount of capsule, epithelial adhesion, anti-phagocytosis and virulence in the type-6B genetic background of *S. pneumoniae.*

## Discussion

The polysaccharide capsule is an outer layer of *S. pneumoniae*, which is essential for immune evasion and virulence of this pathogen. However, due to selection pressure by host immunity, the capsule locus is subjective to extensive sequence variation and antigenic variation through horizontal gene transfer-mediated sequence shuffling^9^,^18^. While it is well recognized that sequence diversity in the coding region of the capsule locus defines serotypes^5^,^8^, the inter-strain sequence variations in the *cps* promoter have been largely ignored thus far. This work represents the first comprehensive investigation into the functional impact of this region. Our sequencing analysis of 225 invasive pneumococcal isolates uncovered several intriguing features in the promoter region of the capsule locus: extensive sequence polymorphisms, differential arrangements of the sequence modules, promiscuous assortments of the promoter with the coding region, and selective preservation of the sequence polymorphisms. Subsequent experiments with unmarked promoter replacement strains in two genetic backgrounds showed that the allelic variations in the *cps* promoter generate profound phenotypic diversity in pneumococcal pathobiology.

Capsule is a common structure of many pathogenic bacteria^19^. At the cellular level, the amount of capsule may be controlled by transcriptional regulators in response to environmental conditions, as exemplified in *Bacillus anthracis*^20^, *E. coli*^21^,^22^,^23^, *Pseudomonas aeruginosa*^24^, and *Streptococcus pyogenes*^25^. At the population level, capsule production can be modulated by reversible sequence insertion/deletion in the capsule genes in *N. meningitidis* group B strains^26^,^27^,^28^, reversible amplification of the capsule locus in the type-b *Haemophilus influenzae*^29^,^30^,^31^, or insertion of the JUMPstart sequence in the capsule promoter of *E. coli*^32^,^33^. The capsule level of pneumococcal serotypes/strains is reversibly affected by spontaneous mutations in the capsule genes^34^,^35^,^36^,^37^, but it remains unknown if the capsule promoter of *S. pneumoniae* is subjective to transcriptional regulation at the cellular level. In this context, our finding in this study represents a unique mechanism for generating strain-to-strain variations in capsule production at the species level by diversifying the sequence and thereby transcriptional capacity of the *cps* promoter.

Our immunoblotting analysis detected significant variations in the level of CPS among the isogenic promoter replacement derivatives in two different strain backgrounds (D39 and ST858). As examples, the promoter variants of types 8, 11A, 23F, and 33F produced up to 7-fold less CPS than the counterparts of other promoter variants (e.g., types 1, 2, 4 and 19A) in the D39 background. This pattern of phenotypic fluctuation was also observed among other promoter variants from different capsular types (inter-type). Further analysis of the type-6B promoter variants in both D39 (type-2) and ST858 (type-6B) also confirmed that the strain-to-strain sequence variations in the *cps* promoter of the same serotype (6B) generate significant diversity in the level of encapsulation. Our finding thus strongly suggests that the variability in the promoter sequence among pneumococcal strains serves as a novel mechanism for quantitative diversification of the capsule at the species level.

As a cover of the pneumococcal cells, the capsule directly interacts with various host cells and molecules for many fitness purposes, such as acquisition of nutrients, adhesion to mucosal epithelium, evasion of molecule- and cell-based bacterial clearance mechanisms, and recruitment of foreign DNA for natural genetic transformation. Electrostatic repulsion conferred by negative charge of the polysaccharide capsule is the critical for proper dispersion of pneumococcal cells and resistance to nonopsonic killing by human neutrophils for optimal colonization and immune evasion^38^,^39^. It is known that different types of the capsule significantly vary in magnitude of negative charge and other properties^39^,^40^,^41^,^42^, which is consistent with uneven prevalence of various pneumococcal serotypes^39^. Multiple studies have also shown that the level of encapsulation significantly affects pneumococcal capability of nasopharyngeal colonization and virulence^38^,^43^,^44^. In this literature context and the profound effect of the promoter sequence variations on the level of encapsulation in this study, we hypothesize that the pneumococci within the same serotypes can behave differently in humans when the same type-specific genes of the capsule locus are naturally driven by different promoter variants. This hypothesis is also supported by our observation that the isogenic *cps* promoter variants of *S. pneumoniae* differed in epithelial adhesion, anti-phagocytosis and virulence. This notion explains why the strains within the same serotypes can differ remarkably in epithelial adhesion, anti-phagocytosis and virulence^17^,^45^,^46^.

The variations among the *cps* promoter variants in the amount of capsule are mostly consistent with their differences in transcriptional capacity as best exemplified by the type-3 and -8 promoter sequences. These two sequences consistently yielded the highest (type-3) and lowest (type-8) values in both the transcription activity and capsule amount. We thus conclude that the differences among the promoter variants in the capsule production are caused by their variations in the transcription of the capsule genes. Our previous study revealed that all of the four sequence modules (IE, RUP, SS, and core promoter) in the *cps* promoter are necessary for the full encapsulation and virulence of strain D39, particularly the SS and core promoter modules^12^. Therefore, sequence variations in any of the sequence modules may alter the transcriptional capacity of the promoter. As illustrated in Fig. 1, there are many nucleotide insertions/deletions and/or multiple arrangements of the sequence modules among the *cps* promoter variants. Although it remains to be defined whether and how each of the sequence changes in the *cps* promoter contributes to the diversification of the *cps* gene transcription and pneumococcal encapsulation, our data have provided a functional foundation for future investigation into pathobiological implications of these promoter variants in pneumococcal fitness and evolution in the different environmental contexts.

Some of the isogenic promoter variants in two genetic backgrounds showed striking differences in virulence in the mouse bacteremia model. As manifested by the type-33F promoter, the bacteremia levels between the isogenic promoter variants can be different by several orders of magnitude. Similar inter-promoter differences in virulence were also observed in the type-6B promoter variants in both the D39 and ST858 backgrounds. These results indicated that the pneumococcal strains within the same serotypes are able to display various levels of virulence and likely other pathobiological traits if their type-specific genes are driven by different promoter variants. Consistent with the importance of the capsule in virulence, the extent of heterogeneity in virulence among the isogenic promoter variants largely parallels with the degree of encapsulation with certain exception. The type-33F promoter replacement strain produced the thinnest capsule and displayed the weakest virulence phenotype among the promoter variants tested thus far. Similarly, other relatively weaker promoters (e.g., types 6B, 8, 11A, 19F, 22F, and 23F) also yielded remarkably low levels of virulence. Because similar promoter sequences are widely shared by the strains of different serotypes, it is reasonable to predict that the pneumococcal strains with these types of promoters belong to the low virulence category. While the capsular types have been associated with the virulence levels of the pneumococci, this work suggests that the promoter type is a new determinant of the capsule-associated virulence phenotype.

The sequence variations in the *cps* promoter consist of minor polymorphisms (e.g., nucleotide replacements, insertions and deletions) and major modular insertions/deletions. While the former is likely to be caused by spontaneous sequence drift, the modular insertions/deletions appears to be originally achieved by acquisition/loss of multiple mobile elements (the RUP and IE modules). The highly mosaic but nonrandom nature of the sequence polymorphisms in the *cps* promoter indicate that most, if not all, of the variations have been maintained and shared by the pneumococcal strains as a function of selective pressure in humans.

Many selective factors may have contributed to the heterogeneity of the *cps* promoter sequence. For example, mucosal epithelial environment may favor the pneumococci with weak *cps* promoter variants, and thereby a relatively thin capsule for efficient colonization. Similarly, it is possible that adaptive immunity specific for the CPS preferentially targets the abundantly encapsulated pneumococci, thus selecting for those with relatively weak *cps* promoter variants. It is thus tempting to postulate the pneumococci with a thin capsule may be better equipped for evasion of the vaccine-induced adaptive immunity and/or vaccine replacement^47^,^48^. Conversely, the pneumococci with the stronger *cps* promoter variants may have advantages in cellular dispersion and evasion of non-opsonic phagocytic killing. Magee *et al*. show that type-3 pneumococcal mutants with a thin capsule (approximately 20% of the parent strain) are able to achieve adequate nasopharyngeal colonization but unable to cause systemic infection^43^. Other studies have shown that quantitative differences in the amount of capsule significantly contribute to the fitness advantages and disadvantages of the opacity phase variants of *S. pneumoniae* in colonization and systemic infection models^38^,^44^,^49^.

**Figure 8:**
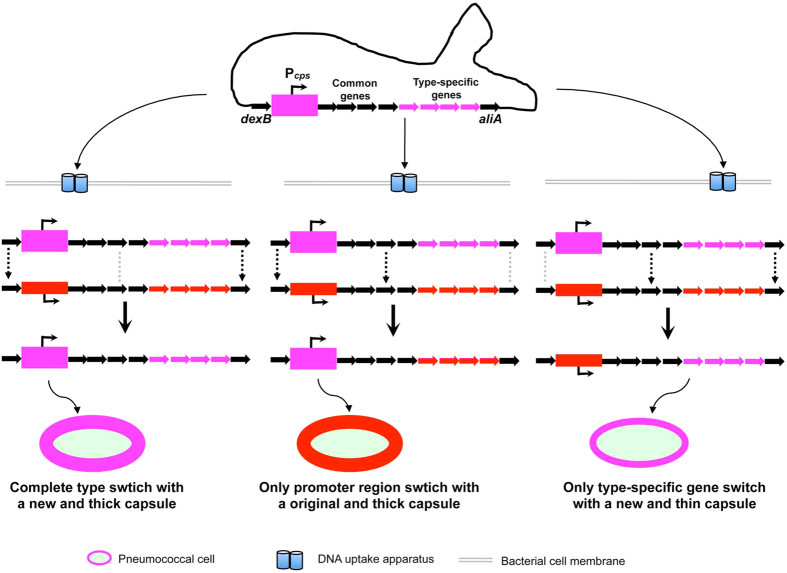
Schematic representation of potential modular recombinations in the capsule locus of *S. pneumoniae*. The abundantly encapsulated pneumococci (represented by the thick rectangle in the promoter region) may lyse and release their chromosomal DNA by autolysis or fratricide. The free DNA can be taken up by the nearby pneumococcal cells through natural genetic transformation, and recombined into the capsule locus of the recipient cells by one of the three modular homologous recombination schemes as indicated by the dashed arrows in each of the scenarios. The double-crossover mediated by the conserved sequences in the up (e.g., *dexB*)- and down (e.g., *aliA*)-stream regions of the locus would result in replacement of the entire capsule locus with the incoming DNA and generate a pneumococcal variant with a new capsule (left panel). Alternative recombination driven by the homologous sequences in the *dexB* region and common capsule genes (e.g., *cpsABCD*) may lead to the replacement of the promoter region but not the type-specific genes, altering the transcription of the capsule genes and level of encapsulation depending on the transcription capacity of the incoming *cps* promoter (middle panel). The last recombination potential occurs in the common genes and the *aliA* region, allowing the recipient cell to capture only the type-specific genes and produce a new capsule from the original promoter (right panel).

*S. pneumoniae* is famous for its ability to modify the genetic content by acquiring foreign DNA through natural genetic transformation^18^,^50^,^51^. The diverse but conserved nature of the *cps* promoter among the invasive isolates in modular arrangements and sequence polymorphisms indicates that the allelic variations in this region is enriched by shuffling the entire or partial sequences across the strains of this species by natural genetic transformation. The lack of strict association between the promoter variants and serotypes also implies that the promoter and coding regions of the capsule locus are able to undergo independent recombination during natural transformation events. It is thus reasonable to envision that sampling of an exogenous DNA molecule containing the complete sequence of the capsule locus may lead to at least three different recombination outcomes (Fig. 8). First, homologous recombination(s) mediated by the common sequences in the *dexB* and *aliA* regions may lead to complete replacement of the original capsule locus with the incoming sequence, and thereby gain a new type of capsule (serotype replacement). Second, the conserved sequences in the *dexB* and common *cps* genes (e.g., *cpsABCD*) may facilitate alternative recombination(s) only in the promoter region. This type of recombination would only alter the thickness but not antigenic/chemical properties of the original capsule. Lastly, homologous recombination(s) may also occur in the type-specific genes, which would retain the original promoter sequence but switch the capsule type. This type of promiscuous assortment between the promoter and coding regions of the capsule locus can generate additional level of phenotypic diversity in different populations (or strains), in addition to the well-know capsule type switch in this species. In the evolutionary perspective, our finding has provided exciting evidence to ultimately uncovering the mystery of pneumococcal resilience in the era of antibiotic and vaccine.

## Materials and Methods

### Bacterial strains, cultivation, and chemical reagents

The bacterial strains used for studying the *cps* promoter variants in this study are listed in Table 1 or Fig. S1. The 225 clinical isolates of *S. pneumoniae* used for sequence analysis of the *cps* promoter variants were obtained from the existing collections of the anonymized bacterial strains in the US CDS and individual Chinese hospitals. All of the isolates were originally isolated from normally sterile sites (e.g. blood, cerebrospinal fluid - CSF) of human patients. The US isolates (n = 47) were provided by the Active Bacterial Core surveillance (ABCs)/Emerging Infections Programs (EIP) Network of the US CDC, Atlanta, USA. The Chinese isolates (n = 178) were prospectively collected by the Fourth Military Medical University affiliated Xijin Hospital, Beijing Children’s Hospital, and China CDC, Beijing. The experiments associated with the use of these strains were carried out in accordance with the principles in the Chinese law for scientific experimentations with human materials and approved by the Institutional Review Board for human studies at Tsinghua University. The serotypes of the isolates were either previously characterized (for the US isolates) or determined by a multiplex PCR-based approach (for the Chinese isolates)^52^. *S. pneumoniae* was cultured in Todd-Hewitt broth with 0.5% yeast extract (THY) or on tryptic soy agar (TSA) plates with 3% defibrinated sheep blood at 37 °C with 5% CO_2_ as described^53^. Growth feature of D39 derivatives were determined in the THY broth as described previously^12^. *Escherichia coli* DH5α was used for constructing the reporter plasmids. All restriction enzymes and chemicals were purchased from New England Biolabs (NEB, Beijing, China), and Sigma-Aldrich (Shanghai, China) respectively unless otherwise stated.

### DNA sequencing analysis

The *cps* promoter region, or the intergenic region between the 3′ *dexB* and 5′ *cpsA*, of all the isolates except for the type-3 strains, was amplified with primers Pr7524 and Pr7525^9^. The promoter region of the type-3 isolates was amplified with primers Pr7662 and Pr8138. The sequences of the PCR products were determined by the ABI sequencer and analyzed by BLAST and relevant components of the DNAstar’s Lasergene package (version 10). The GenBank accession numbers for the *cps* promoter sequences used in the promoter replacement experiments are listed in Table 1.

### Construction of transcriptional reporter constructs and luciferase assay

Luciferase reporter constructs of the *cps* promoter were generated in pIB166 shuttle plasmid as described^12^. The sequence between the stop codon of *dexB* and ribosomal binding site of *cpsA* was amplified from the genomic DNA of each target pneumococcal strain, and cloned in the ApaI/BamHI site of pTH3932^12^, a derivative of pIB166^54^ containing the promoterless firefly luciferase gene. The insert sequence in each recombinant plasmid was verified by DNA sequencing. The resulting plasmids and primers/source strains used to prepare them are listed in Table 1. Luciferase assay was performed as described previously^12^. Briefly, a pellet from 100 μl original culture was suspended in 400 μl phosphate-buffered saline (PBS), pH 7.4; a fraction out of the suspension (20 μl) was placed into a Corning NBS 96-well white plate with clear bottom. Subsequently, luciferase substrate (100 μl; Promega, Madison, WI) was added to each well to allow the monitoring of luminescence. Each reporter construct was tested at least three separate times. The results of representative experiments are presented as means of three replicates ± standard errors.

### Construction of pneumococcal mutants

All unmarked deletions and sequence replacements in *S. pneumoniae* strains were carried out with the Janus cassette (JC)-mediated counter selection as described previously^55^. Strains D39s (an streptomycin-resistant derivative of D39), TH4702 (D39s-ΔP*cps*::JC, a *cps*-promoter JC-replacement derivative of D39s), TH4525 (TH4702-ΔP*cps*, a *cps*-promoter unmarked deletion derivative of TH4702) were previously constructed^12^. The unmarked *cps* promoter replacement strains were generated in D39s or ST858s, a streptomycin-resistant derivative of type-6B strain ST858. ST858 was originally isolated from the blood of a patient in the US and obtained from the US CDC from the Active Bacterial Core surveillance (ABCs)/Emerging Infections Programs (EIP) Network of the US CDC. ST858s was generated with an amplicon of the *rpsL1*-containing sequence from strain ST588^55^, and used to generate the JC knock-in mutant TH7180 (ST858s-ΔP*cps*::JC) by replacement of the *cps* promoter region (sequence between the *dexB* stop codon and *cps6BA* start codon) in ST858s with JC through an amplicon from genomic DNA of TH4702 with primers Pr7339/Pr7344. The unmarked promoter replacement strains were prepared in the D39 and ST858 backgrounds by amplifying the flanking sequences of the *cps* promoter region from genomic DNA of each strain with the same primer set: Pr7339/Pr7340 and Pr7343/Pr7344, linking the PCR products to each exogenous *cps* promoter amplicon by fusion PCR using Primers Pr7339 and Pr7344, transforming TH4702 or TH7180 with target fusion PCR products, and selected/screened for streptomycin-resistant and kanamycin-sensitive transformants. The exogenous *cps* promoter sequences were individually amplified from genomic DNA samples of the source strains with strain-specific primers as described in Table S3. All of the mutated loci in the resulting pneumococcal mutants were verified by DNA sequencing.

### RNA extraction and quantitative real-time reverse transcriptase PCR (qRT-PCR)

Total pneumococcal RNA was purified using an RNAprep pure Cell/Bacteria Kit (TianGen, Beijing, China) according to the supplier’s instructions. The mRNA level of the *cps* genes was quantified by quantitative RT-PCR (qRT-PCR) as described^12^. The pneumococcal *rplI* gene encoding the ribosomal protein RplI was amplified with primers Pr7709 and Pr7710 as an internal control. The transcripts of the *cps2A/6BA* and *cps2E/6BE* genes were detected with primer pairs Pr7705/Pr7706 and Pr7707/Pr7708, respectively. Each qRT-PCR experiment was repeated at least three times. The results of representative experiments are presented as means of three replicates ± standard errors.

### Anti-CPS antibodies and immunoblotting

Pneumococcal CPS was detected by immunoblotting as described previously^12^. The type-2 or -6B capsular polysaccharide in D39 and ST858 derivatives was semi-quantified with a rabbit antiserum against the type-2 CPS (1:20,000 dilution) from Statens Serum Institut (Copenhagen, Denmark) or a self-prepared rabbit antiserum against the CPSs (types 4, 6B, 9V, 14, 18C, 19F, and 23F) (1:100 dilution) in the pneumococcal PCV7 vaccine. Anti-PCV7 serum was prepared in New Zealand white rabbits (Vital River, Beijing, China) by subcutaneous immunization with the PCV vaccine according to the standard protocol^56^. The animal works associated with the antibody production in rabbits were in accordance with the principles in the Chinese law on the humane use of animals for scientific experimentations, and were approved by the Institutional Animal Care and Use Committee in Tsinghua University. The bacterial cultures were pelleted by centrifugation and resuspended to an OD_620_ of 0.4 in phosphate-buffered saline (PBS) and 2-fold dilution before being used for CPS immunoblotting. The CPS-binding activities of the primary antibodies were detected by peroxidase-conjugated goat anti-rabbit IgG (H+L) (Bio-Rad, 1:10,000 dilution) and the ClarityTM Western ECL reagent (Bio-Rad). The ImageJ software (ImageJ 1.47v; National Institutes of Health) was used to digitize the amount of pneumococcal CPS on 2X, 4X, 8X and 16X dilutions spots on the basis of its chemiluminescence intensity level. The results of representative experiments are presented as means of three replicates ± standard errors. The error bars are means of the detectable spots for each sample.

### Bacterial adhesion to epithelial cells and phagocytosis

Pneumococcal adhesion to host cells was determined with the confluent monolayers of human lung alveolar epithelial cell line A549 in 24-well plates as described in our previous study^12^. Each monolayer was infected with 10^7^ colony-forming units (CFUs) of pneumococci. Phagocytosis of pneumococcal was determined with RAW264.7 murine macrophage cells^57^. Bacteria were incubated with RAW264.7 cells for 2 h, followed by incubating 1 h to kill extracellular bacteria with Penicillin (20 μg/ml) and gentamycin (400 μg/ml), extensive washing and lysis of the cells. The adherent or invasion bacteria were quantified by plating the cell lysates on TSA blood plates and counting the CFU. Each adhesion or phagocytosis experiment was repeated at least three times. The results of representative experiments are presented as means of four replicates ± standard errors.

### Pneumococcal virulence test

The virulence capacity of pneumococcal strains was assessed in a murine bacteremia model as previously described^12^. The animal works associated with pneumococcal infection in mice were conducted in accordance with the principles in the Chinese law on the humane use of animals for scientific experimentations, and were approved by the Institutional Animal Care and Use Committee in Tsinghua University. Briefly, groups of 10 female CD1 mice (6–8 weeks old, Vital River) were infected by intraperitoneal inoculation with approximately 500 CFUs per mouse for each co-infection of D39 and its derivative (~250 CFUs each) or 10^6^ CFUs per mouse for the corresponding experiment with ST858 and its derivative (~5 × 10^5^ CFUs each). The viable pneumococci in the bloodstream of each mouse were quantified by plating the blood sample on TSA agar dishes with (mutant) or without (mutant plus wild type) streptomycin selection 21 h post inoculation for enumeration of colonies. The level of attenuation was expressed as a competitive index (CI), which was defined as the output CFUs ratio (mutant/wild type) divided by the input CFU ratio (mutant/wild type) as described^12^. This factor was considered in calculation of the final CI values. When only the wild-type pneumococci (no mutant) were recovered from a mouse in the co-infection experiments, a value of 1 was given for the mutant output for the convenience of data presentation in common logarithms.

### Statistical analysis

Statistical significance of the data from qRT-PCR, luciferase, and immunoblotting was determined by two-tailed unpaired Student’s *t* test. The data from animal infection and cell experiments were analyzed by Wilcoxon signed-rank and one-way analysis of variance (ANOVA) tests, respectively. Significant differences are defined by *p* values <0.05 (*), <0.01 (**), and <0.001(***).

## Additional Information

**How to cite this article**: Wen, Z. *et al*. Allelic Variation of the Capsule Promoter Diversifies Encapsulation and Virulence In *Streptococcus pneumoniae*. *Sci. Rep.* **6**, 30176; doi: 10.1038/srep30176 (2016).

## Supplementary Material

Supplementary Information

## Figures and Tables

**Table 1:** The promoter variants of the pneumococcal capsule locus used in this study.

Promoter/serotype ID	Relevant strain/plasmid	Amplification primers	Source strain	Genbank accession
1	TH5222, pTH4159	Pr7524/Pr7525 Pr6269/Pr6274	TH2927	KU295440
2	D39s, pTH3937		D39	AF026471
3	TH5123, pTH4856	Pr7662/Pr8138 Pr6269/Pr7622	ST868	KU295427
4	TH4766, pTH3944	Pr7524/Pr7525 Pr6269/Pr6274	TIGR4	AE005672
6B-1	TH4767, TH7331, pTH4721	Pr7524/Pr7525 Pr6269/Pr7322	TH2882	KU295438
6B-2	TH4772, TH7181	Pr7524/Pr7525	TH2787	KU295442
6B-3	TH4773, TH7239	Pr7524/Pr7525	TH2925	KU295444
6B-4	TH4774, TH7242	Pr7524/Pr7525	TH2801	KU295443
6B-5	TH7052, TH7181	Pr7524/Pr7525	ST858	KU295441
7F	pTH3955	Pr6269/Pr6271	ST898	KU295434
8	TH5121, pTH4712	Pr7524/Pr7525 Pr6269/Pr6213	TH2864	KU295437
9N	pTH4857	Pr6269/Pr7622	TH4848	CR931647
9V-1	pTH3948	Pr6269/Pr6270	ST883	KU295432
9V-2	None	None	TH2774	KU324512
10A	pTH4718	Pr6269/Pr7323	ST860	KU295425
11A	TH5217, pTH4858	Pr7524/Pr7525 Pr6269/Pr7324	TH4849	CR931653
12F	pTH4723	Pr6269/Pr7350	ST895	KU295433
14-1	TH4768, pTH4724	Pr7524/Pr7525 Pr6269/Pr7321	TH2889	KU295439
14-2	None	None	TH2938	KU324513
15B	pTH4714	Pr6269/Pr6270	TH2580	KU295435
16F	N/A	N/A	TH2811	KU324514
17F	pTH4859	Pr6269/Pr7324	TH4850	CR931670
18C	TH4769, pTH4239	Pr7524/Pr7525 Pr6269/Pr6273	ST882	KU295431
19A	TH5214, pTH3946	Pr7524/Pr7525 Pr6269/Pr6270	ST873	KU295429
19F-1	TH4770, pTH3954	Pr7524/Pr7525 Pr6269/Pr6270	Taiwan19F-14	NC012469
19F-2	None	None	TH2802	KU324515
20	pTH4719	Pr6269/Pr6270	TH2592	KU295436
22F	TH5207, pTH4728	Pr7524/Pr7525 Pr6269/Pr7325	ST872	KU295428
23F-1	TH7054, pTH3953	Pr7524/Pr7525 Pr6269/Pr6270	ST862	KU295426
23F-2	None	None	TH2881	KU324516
33F	TH4771, pTH4706	Pr7524/Pr7525 Pr6269/Pr7324	ST874	KU295430
35B	None	None	TH2733	KU295447

## References

[b1] Musher D. M. In Principles and Practice of Infectious Diseases Vol. 2 (eds Mandell G. L., Bennett J. E. & Dolin R. D. ) 2623–2642 (Elsevier Churchill Livingstone, 2010).

[b2] O’Brien K. L. . Burden of disease caused by Streptococcus pneumoniae in children younger than 5 years: global estimates. Lancet 374, 893–902, 10.1016/S0140-6736(09)61204-6 (2009). 19748398

[b3] Walker C. L. . Global burden of childhood pneumonia and diarrhoea. Lancet 381, 1405–1416, 10.1016/S0140-6736(13)60222-6 (2013). 23582727 PMC7159282

[b4] Avery O. T., MacLeod C. M. & McCarty M. Studies on the chemical nature of the substance inducing transformation of pneumococcal types. J. Exp. Med. 79, 137–158 (1944). 19871359 10.1084/jem.79.2.137 PMC2135445

[b5] Yother J. Capsules of Streptococcus pneumoniae and other bacteria: paradigms for polysaccharide biosynthesis and regulation. Annu Rev Microbiol 65, 563–581, 10.1146/annurev.micro.62.081307.162944 (2011). 21721938

[b6] Nahm M. H. & Katz J. In Fundamental Immunology (ed William E. P. ) Ch. 41, 1001–1015 (Lippincott-Raven Publishers, 2012).

[b7] Lee C. J., Banks S. D. & Li J. P. Virulence, immunity, and vaccine related to Streptococcus pneumoniae. Crit Rev Microbiol 18, 89–114, 10.3109/10408419109113510 (1991). 1930677

[b8] Geno K. A. . Pneumococcal Capsules and Their Types: Past, Present, and Future. Clin Microbiol Rev 28, 871–899, 10.1128/CMR.00024-15 (2015). 26085553 PMC4475641

[b9] Bentley S. D. . Genetic analysis of the capsular biosynthetic locus from all 90 pneumococcal serotypes. PLoS Genet 2, e31, 10.1371/journal.pgen.0020031 (2006). 16532061 PMC1391919

[b10] Llull D., Munoz R., Lopez R. & Garcia E. A single gene (tts) located outside the cap locus directs the formation of Streptococcus pneumoniae type 37 capsular polysaccharide. Type 37 pneumococci are natural, genetically binary strains. J Exp Med 190, 241–251, 10.1084/jem.190.2.241 (1999). 10432287 PMC2195575

[b11] Iannelli F., Pearce B. J. & Pozzi G. The type 2 capsule locus of Streptococcus pneumoniae. J Bacteriol 181, 2652–2654 (1999). 10198036 10.1128/jb.181.8.2652-2654.1999 PMC93698

[b12] Wen Z. . Sequence elements upstream of the core promoter are necessary for full transcription of the capsule gene operon in Streptococcus pneumoniae strain D39. Infect Immun 83, 1957–1972, 10.1128/IAI.02944-14 (2015). 25733517 PMC4399061

[b13] Moscoso M. & Garcia E. Transcriptional regulation of the capsular polysaccharide biosynthesis locus of streptococcus pneumoniae: a bioinformatic analysis. DNA Res 16, 177–186, 10.1093/dnares/dsp007 (2009). 19429668 PMC2695774

[b14] Shainheit M. G., Mule M. & Camilli A. The core promoter of the capsule operon of Streptococcus pneumoniae is necessary for colonization and invasive disease. Infect Immun 82, 694–705, 10.1128/IAI.01289-13 (2014). 24478084 PMC3911406

[b15] Feldman C. & Anderson R. Review: Current and new generation pneumococcal vaccines. J Infect 69, 309–325, 10.1016/j.jinf.2014.06.006 (2014). 24968238

[b16] Lanie J. A. . Genome sequence of Avery’s virulent serotype 2 strain D39 of Streptococcus pneumoniae and comparison with that of unencapsulated laboratory strain R6. J Bacteriol 189, 38–51, 10.1128/JB.01148-06 (2007). 17041037 PMC1797212

[b17] Ring A., Weiser J. N. & Tuomanen E. I. Pneumococcal trafficking across the blood-brain barrier. Molecular analysis of a novel bidirectional pathway. J. Clin. Invest 102, 347–360, 10.1172/JCI2406 (1998). 9664076 PMC508893

[b18] Chewapreecha C. . Dense genomic sampling identifies highways of pneumococcal recombination. Nature genetics 46, 305–309, 10.1038/ng.2895 (2014). 24509479 PMC3970364

[b19] Wen Z. & Zhang J.-R. In Molecular Medical Microbiology Vol. 1 (eds Tang Y.-W. . ) Ch. 3, 33–52 (Academic Press, 2015).

[b20] Drysdale M., Bourgogne A. & Koehler T. M. Transcriptional analysis of the Bacillus anthracis capsule regulators. J Bacteriol 187, 5108–5114, 10.1128/JB.187.15.5108-5114 (2005). 16030203 PMC1196023

[b21] Gottesman S. & Stout V. Regulation of capsular polysaccharide synthesis in Escherichia coli K12. Molecular microbiology 5, 1599–1606, 10.1111/j.1365-2958.1991.tb01906.x (1991). 1943696

[b22] Torres-Cabassa A. S. & Gottesman S. Capsule synthesis in Escherichia coli K-12 is regulated by proteolysis. J Bacteriol 169, 981–989 (1987). 3029041 10.1128/jb.169.3.981-989.1987 PMC211890

[b23] Majdalani N. & Gottesman S. The Rcs phosphorelay: a complex signal transduction system. Annu Rev Microbiol 59, 379–405, 10.1146/annurev.micro.59.050405.101230 (2005). 16153174

[b24] Bayer A. S., Eftekhar F., Tu J., Nast C. C. & Speert D. P. Oxygen-dependent up-regulation of mucoid exopolysaccharide (alginate) production in Pseudomonas aeruginosa. Infection and immunity 58, 1344–1349 (1990). 2139011 10.1128/iai.58.5.1344-1349.1990 PMC258630

[b25] Wessels M. R. In Gram-Positive Pathogens (eds Fischetti V. A. . ) 37–46 (ASM Press, 2006).

[b26] Hilse R., Hammerschmidt S., Bautsch W. & Frosch M. Site-specific insertion of IS1301 and distribution in Neisseria meningitidis strains. J Bacteriol 178, 2527–2532 (1996). 8626318 10.1128/jb.178.9.2527-2532.1996 PMC177975

[b27] Hammerschmidt S. . Modulation of cell surface sialic acid expression in Neisseria meningitidis via a transposable genetic element. EMBO J 15, 192–198 (1996). 8598202 PMC449931

[b28] Hammerschmidt S. . Capsule phase variation in Neisseria meningitidis serogroup B by slipped-strand mispairing in the polysialyltransferase gene (siaD): correlation with bacterial invasion and the outbreak of meningococcal disease. Mol Microbiol 20, 1211–1220, 10.1111/j.1365-2958.1996.tb02641.x (1996). 8809773

[b29] Kroll J. S., Loynds B. M. & Moxon E. R. The Haemophilus influenzae capsulation gene cluster: a compound transposon. Mol Microbiol 5, 1549–1560, 10.1111/j.1365-2958.1991.tb00802.x (1991). 1664907

[b30] Corn P. G., Anders J., Takala A. K., Kayhty H. & Hoiseth S. K. Genes involved in Haemophilus influenzae type b capsule expression are frequently amplified. J Infect Dis 167, 356–364, 10.1093/infdis/167.2.356 (1993). 8421169

[b31] Noel G. J., Brittingham A., Granato A. A. & Mosser D. M. Effect of amplification of the Cap b locus on complement-mediated bacteriolysis and opsonization of type b Haemophilus influenzae. Infection and immunity 64, 4769–4775 (1996). 8890238 10.1128/iai.64.11.4769-4775.1996 PMC174444

[b32] Xue P., Corbett D., Goldrick M., Naylor C. & Roberts I. S. Regulation of expression of the region 3 promoter of the Escherichia coli K5 capsule gene cluster involves H-NS, SlyA, and a large 5′ untranslated region. J Bacteriol 191, 1838–1846, 10.1128/JB.01388-08 (2009). 19114478 PMC2648368

[b33] Hobbs M. & Reeves P. R. The JUMPstart sequence: a 39 bp element common to several polysaccharide gene clusters. Molecular microbiology 12, 855–856, 10.1111/j.1365-2958.1994.tb01071.x (1994). 8052136

[b34] Waite R. D., Struthers J. K. & Dowson C. G. Spontaneous sequence duplication within an open reading frame of the pneumococcal type 3 capsule locus causes high-frequency phase variation. Mol Microbiol 42, 1223–1232, 10.1046/j.1365-2958.2001.02674.x (2001). 11886554

[b35] Waite R. D., Penfold D. W., Struthers J. K. & Dowson C. G. Spontaneous sequence duplications within capsule genes cap8E and tts control phase variation in Streptococcus pneumoniae serotypes 8 and 37. Microbiology 149, 497–504, 10.1099/mic.0.26011-0 (2003). 12624211

[b36] Schaffner T. O. . A point mutation in cpsE renders Streptococcus pneumoniae nonencapsulated and enhances its growth, adherence and competence. BMC microbiology 14, 210, 10.1186/s12866-014-0210-x (2014). 25163487 PMC4243769

[b37] Shainheit M. G., Valentino M. D., Gilmore M. S. & Camilli A. Mutations in pneumococcal cpsE generated via *in vitro* serial passaging reveal a potential mechanism of reduced encapsulation utilized by a conjunctival isolate. Journal of bacteriology 197, 1781–1791, 10.1128/JB.02602-14 (2015). 25777672 PMC4402401

[b38] Nelson A. L. . Capsule enhances pneumococcal colonization by limiting mucus-mediated clearance. Infect Immun 75, 83–90, 10.1128/IAI.01475-06 (2007). 17088346 PMC1828419

[b39] Li Y., Weinberger D. M., Thompson C. M., Trzcinski K. & Lipsitch M. Surface charge of Streptococcus pneumoniae predicts serotype distribution. Infection and immunity 81, 4519–4524, 10.1128/IAI.00724-13 (2013). 24082068 PMC3837974

[b40] Melin M. . Streptococcus pneumoniae capsular serotype 19F is more resistant to C3 deposition and less sensitive to opsonophagocytosis than serotype 6B. Infect Immun 77, 676–684, 10.1128/IAI.01186-08 (2009). 19047408 PMC2632042

[b41] Melin M. . Serotype-related variation in susceptibility to complement deposition and opsonophagocytosis among clinical isolates of Streptococcus pneumoniae. Infect Immun 78, 5252–5261, 10.1128/IAI.00739-10 (2010). 20855517 PMC2981318

[b42] Melin M., Trzcinski K., Meri S., Kayhty H. & Vakevainen M. The capsular serotype of Streptococcus pneumoniae is more important than the genetic background for resistance to complement. Infect Immun 78, 5262–5270, 10.1128/IAI.00740-10 (2010). 20855513 PMC2981297

[b43] Magee A. D. & Yother J. Requirement for capsule in colonization by Streptococcus pneumoniae. Infect Immun 69, 3755–3761, 10.1128/IAI.69.6.3755-3761.2001 (2001). 11349040 PMC98386

[b44] Kim J. O. & Weiser J. N. Association of intrastrain phase variation in quantity of capsular polysaccharide and teichoic acid with the virulence of Streptococcus pneumoniae. J Infect Dis 177, 368–377, 10.1086/514205 (1998). 9466523

[b45] Frazao N. . Virulence potential and genome-wide characterization of drug resistant Streptococcus pneumoniae clones selected *in vivo* by the 7-valent pneumococcal conjugate vaccine. PloS one 8, e74867, 10.1371/journal.pone.0074867 (2013). 24069360 PMC3777985

[b46] Mizrachi Nebenzahl Y. . Virulence of Streptococcus pneumoniae may be determined independently of capsular polysaccharide. FEMS Microbiol Lett 233, 147–152, doi: http://dx.doi.org/10.1016/j.femsle.2004.02.003 (2004). 15043881 10.1016/j.femsle.2004.02.003

[b47] Croucher N. J. . Population genomics of post-vaccine changes in pneumococcal epidemiology. Nature genetics 45, 656–663, 10.1038/ng.2625 (2013). 23644493 PMC3725542

[b48] Weinberger D. M., Malley R. & Lipsitch M. Serotype replacement in disease after pneumococcal vaccination. Lancet 378, 1962–1973, 10.1016/S0140-6736(10)62225-8 (2011). 21492929 PMC3256741

[b49] Weiser J. N. In Gram-Positive Pathogens (eds Fischetti V. A. . ) 268–274 (American Society for Microbiology Press, 2006).

[b50] Hanage W. P., Fraser C., Tang J., Connor T. R. & Corander J. Hyper-recombination, diversity, and antibiotic resistance in pneumococcus. Science 324, 1454–1457, 10.1126/science.1171908 (2009). 19520963

[b51] Croucher N. J. . Rapid pneumococcal evolution in response to clinical interventions. Science 331, 430–434, 10.1126/science.1198545 (2011). 21273480 PMC3648787

[b52] Pai R., Gertz R. E. & Beall B. Sequential multiplex PCR approach for determining capsular serotypes of Streptococcus pneumoniae isolates. J Clin Microbiol 44, 124–131, 10.1128/JCM.44.1.124-131.2006 (2006). 16390959 PMC1351965

[b53] Chen H. . Genetic requirement for pneumococcal ear infection. PLoS One 3, e2950, 10.1371/journal.pone.0002950 (2008). 18670623 PMC2593789

[b54] Biswas I., Jha J. K. & Fromm N. Shuttle expression plasmids for genetic studies in Streptococcus mutans. Microbiology 154, 2275–2282, 10.1099/mic.0.2008/019265-0 (2008). 18667560 PMC4110107

[b55] Lu L., Ma Y. & Zhang J. R. Streptococcus pneumoniae recruits complement factor H through the amino terminus of CbpA. J Biol Chem. 281, 15464–15474, 10.1074/jbc.M602404200 (2006). 16597618

[b56] Harlow E. & Lane D. Using antibodies: A laboratory manual. (Cold Spring Harbor Laboratory Press, 1999).

[b57] Zhang J. R. . The polymeric immunoglobulin receptor translocates pneumococci across human nasopharyngeal epithelial cells. Cell 102, 827–837, 10.1016/S0092-8674(00)00071-4 (2000). 11030626

[b58] Thompson J. D., Higgins D. G. & Gibson T. J. CLUSTAL W: improving the sensitivity of progressive multiple sequence alignment through sequence weighting, position-specific gap penalties and weight matrix choice. Nucleic Acids Res 22, 4673–4680, 10.1093/nar/22.22.4673 (1994). 7984417 PMC308517

