# Supplementary material for: Allelic Variation of the Capsule Promoter Diversifies Encapsulation and Virulence In Streptococcus pneumoniae
Source: Sci Rep. 2016 Jul 28;6:30176. doi: 10.1038/srep30176 (PMC4964562; doi:10.1038/srep30176)
Supplement: Supplementary Information [file srep30176-s1.doc]

**Allelic Variation of the Capsule Promoter Diversifies Encapsulation**

**and Virulence In *Streptococcus pneumoniae***

Zhensong Wen^1#^, Yanni Liu^1#^, Fen Qu^2^, and Jing-Ren Zhang^1,3^*

^1^Center for Infectious Disease Research, School of Medicine, Tsinghua University, Beijing 100084, China; ^2^The Center of Clinical Diagnosis, 302 Hospital of PLA, Beijing, China; ^3^Collaborative Innovation Center for Biotherapy, State Key Laboratory of Biotherapy and Center, West China Hospital, West China Medical School, Sichuan University, Chengdu, China

**^#^**These authors contributed equally to this work.

*Correspondence to: Jing-Ren Zhang, Center for Infectious Disease Research, School of Medicine, Tsinghua University, Beijing 100084, China; Phone: 86-10-6279-5892; Fax: 86-10-6279-5892; e-mail: [zhanglab@tsinghua.edu.cn](mailto:zhanglab@tsinghua.edu.cn)

**Supplemental Figures and Tables**

**Figure S1.** The sequence variations of the *cps* promoter among the 21 IPD serotypes as determined by the Cluster W method. (**A**) Phylogenetic tree reflecting the sequence homology among the full promoter sequences; (**B**) Relative closeness of the full promoter sequences.


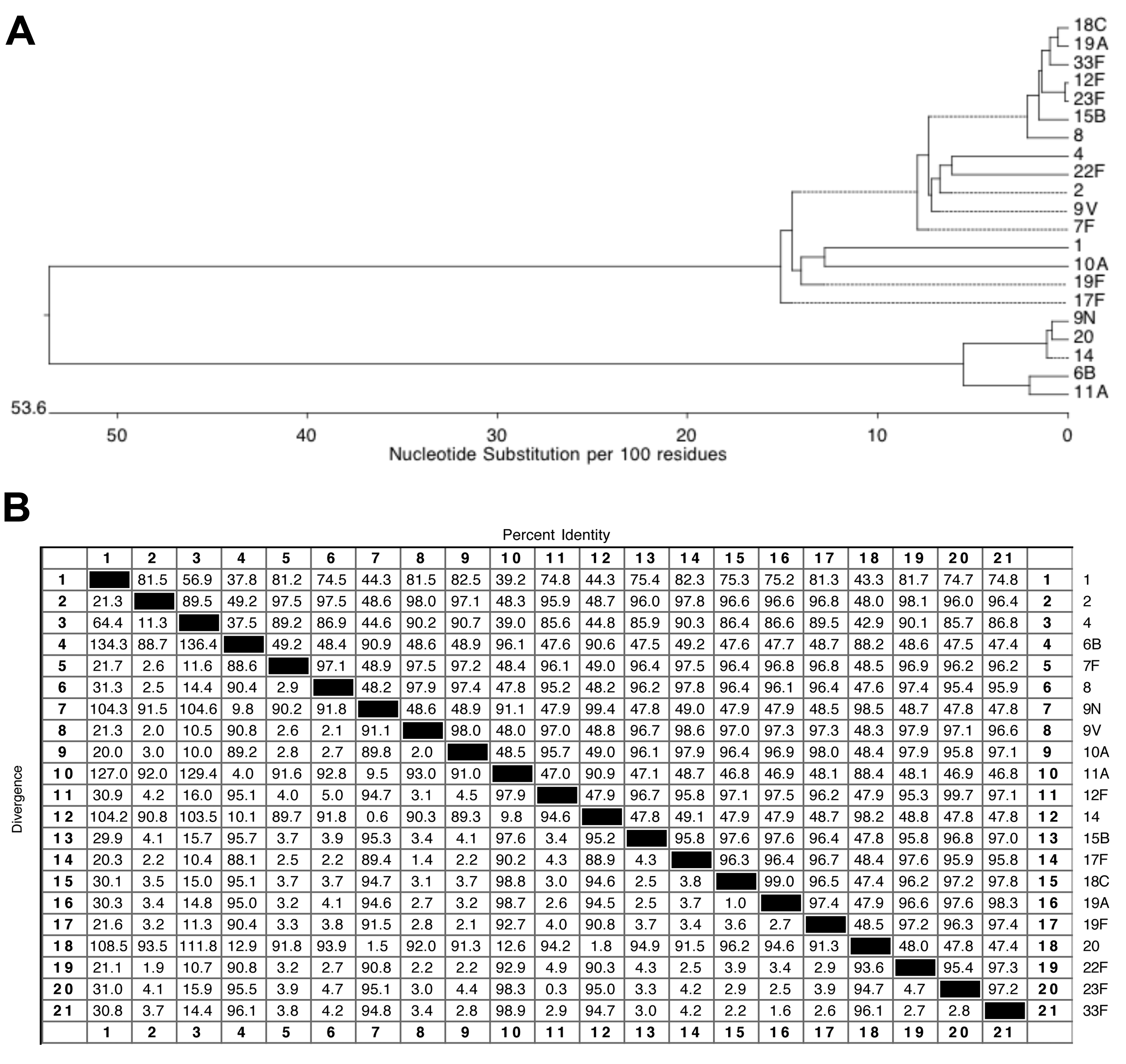


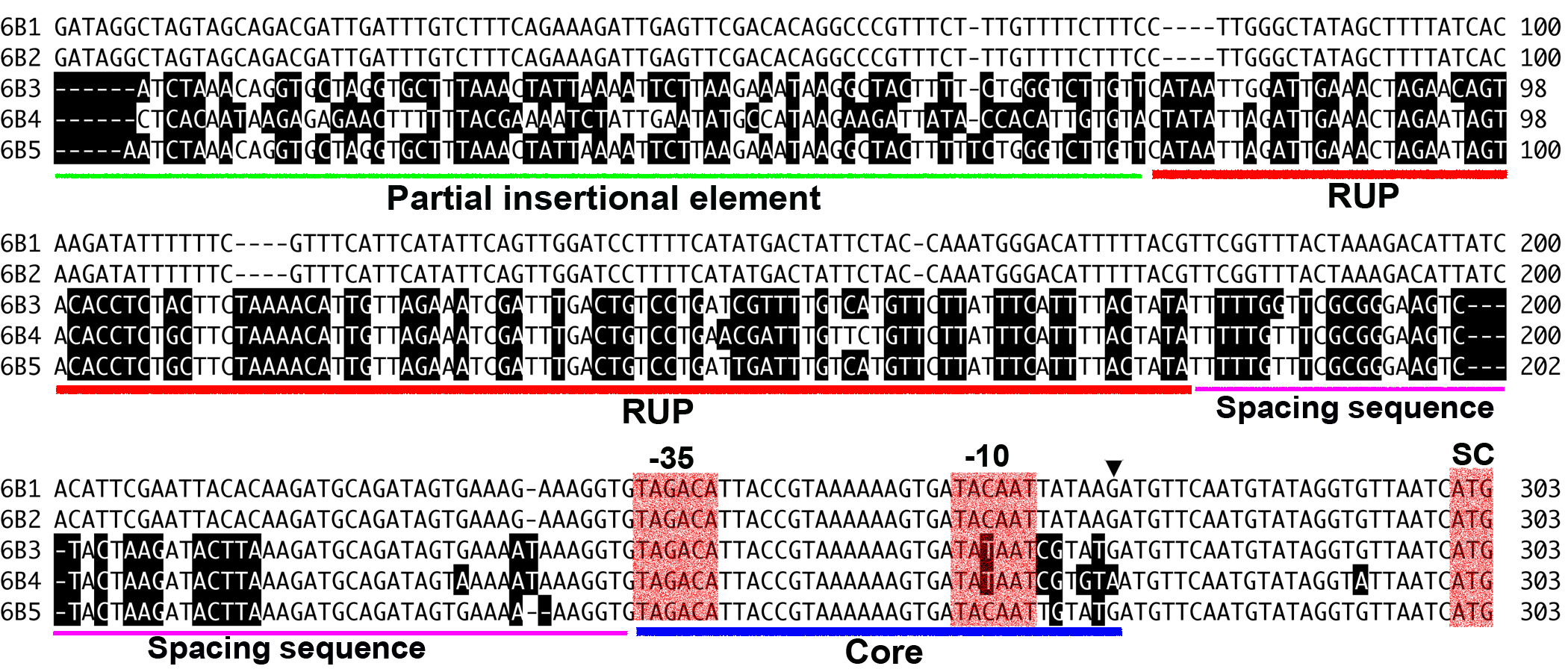
**Figure S2.** Alignment of the *cps* promoter sequences from five type-6B strains illustrated in Fig. 1B.

**Figure S3.** Alignment of the *cps* promoter sequences from types 6B, 9V, 16F, 19F, and 23F strains as illustrated in Fig. 1C.

**
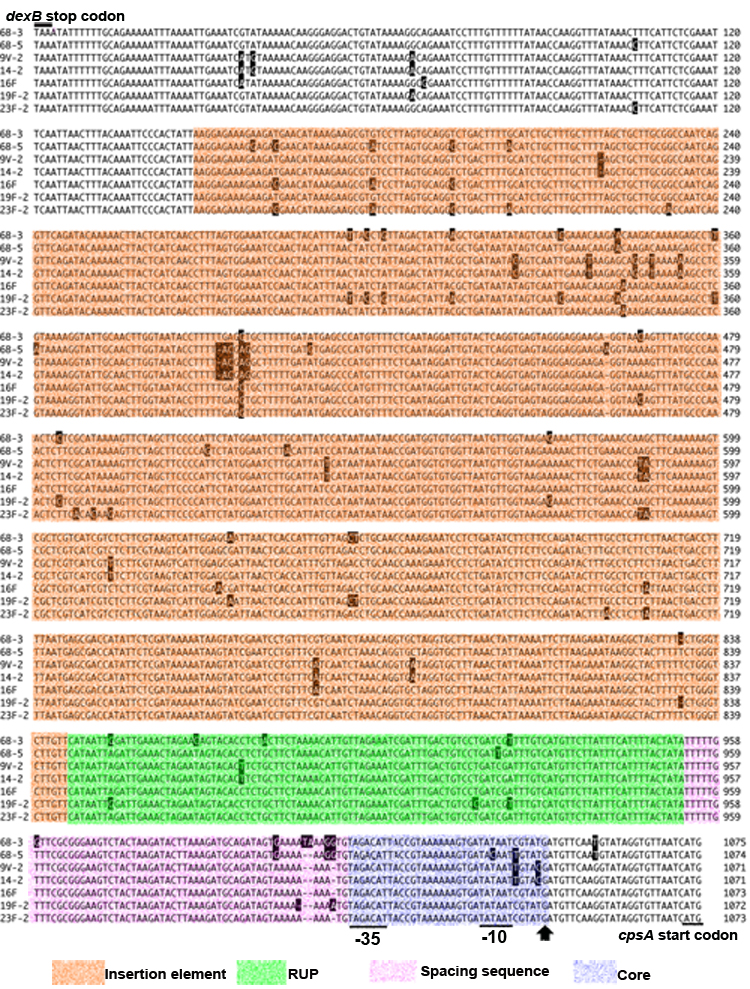
**

**Figure S4.** The qRT-PCR readouts of the *cps2E* and *cps6BE* transcripts from the promoter replacement D39 and ST858 derivatives.

**
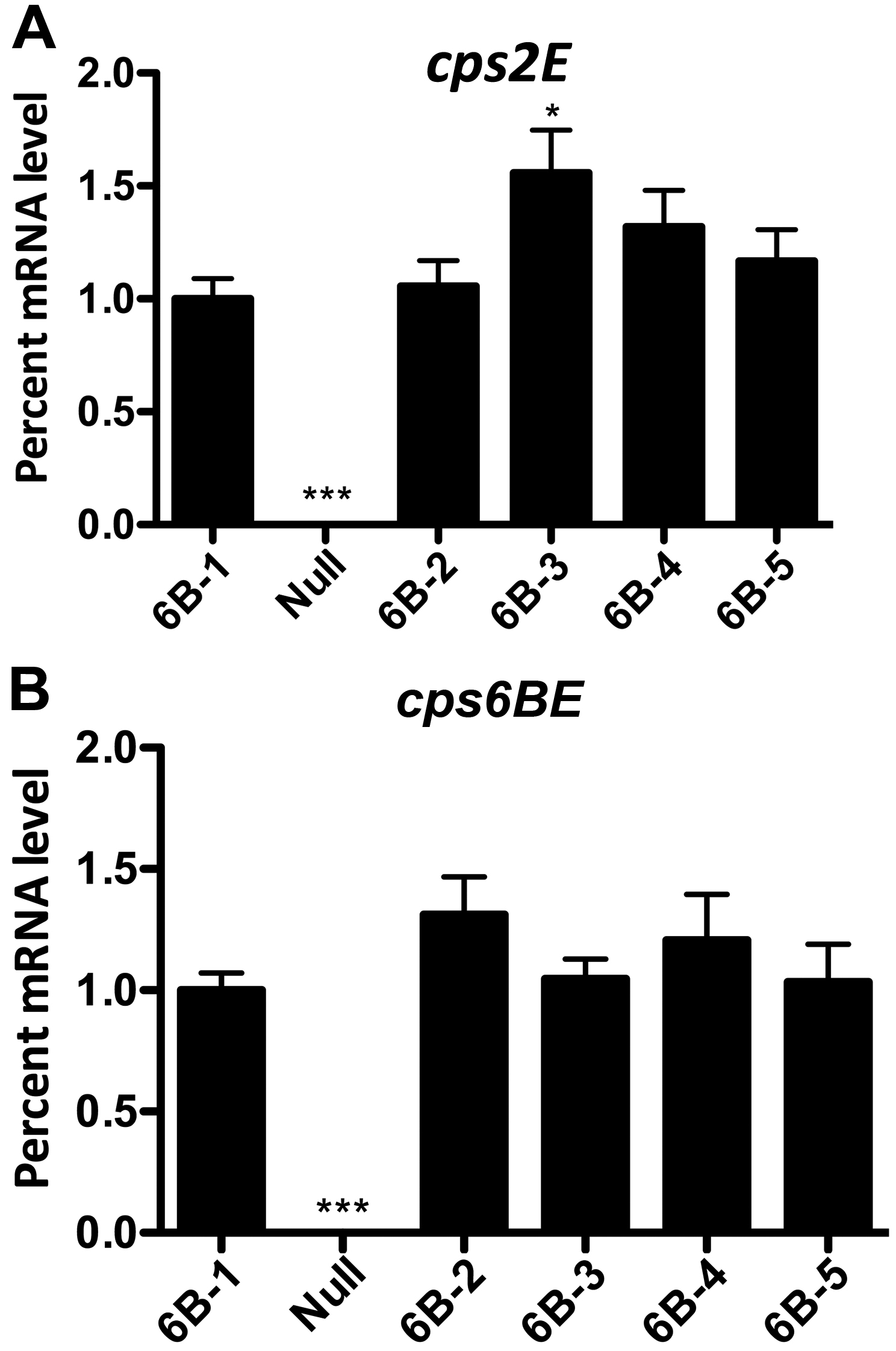
**

**Table S1. Bacterial strains** used in this study

| **Strains** | **Description^a^** | **Reference or source^b^** |
| --- | --- | --- |
| D39 | *S. pneumoniae* strain D39, serotype 2, encapsulated | NCTC |
| D39s | D39 derivative, *rpsL1,* Sm^R^ | (12) |
| TIGR4 | *S. pneumoniae*, serotype 4, encapsulated | ATCC |
| Taiwan19F-14 | *S. pneumoniae,* serotype 19F, encapsulated | ATCC |
| ST858 | *S. pneumoniae,* serotype 6B, encapsulated | CDC |
| ST860 | *S. pneumoniae,* serotype 10A, encapsulated | CDC |
| ST862 | *S. pneumoniae,* serotype 23F, encapsulated | CDC |
| ST868 | *S. pneumoniae,* serotype 3, encapsulated | CDC |
| ST872 | *S. pneumoniae,* serotype 22F, encapsulated | CDC |
| ST873 | *S. pneumoniae,* serotype 19A, encapsulated | CDC |
| ST874 | *S. pneumoniae,* serotype 33F, encapsulated | CDC |
| ST882 | *S. pneumoniae,* serotype 18C, encapsulated | CDC |
| ST883 | *S. pneumoniae,* serotype 9V, encapsulated | CDC |
| ST895 | *S. pneumoniae,* serotype 12F, encapsulated | CDC |
| ST898 | *S. pneumoniae,* serotype 7F, encapsulated | CDC |
| TH2580 | *S. pneumoniae,* serotype 15B, encapsulated | CDC |
| TH2881 | *S. pneumoniae,* serotype 23F, encapsulated | CDC |
| TH2592 | *S. pneumoniae,* serotype 20, encapsulated | CDC |
| TH2787 | *S. pneumoniae,* serotype 6B, encapsulated | CDC |
| TH2801 | *S. pneumoniae,* serotype 6B, encapsulated | CDC |
| TH2864 | *S. pneumoniae,* serotype 8, encapsulated | CDC |
| TH2882 | *S. pneumoniae,* serotype 6B, encapsulated | CDC |
| TH2889 | *S. pneumoniae,* serotype 14, encapsulated | CDC |
| TH2925 | *S. pneumoniae,* serotype 6B, encapsulated | CDC |
| TH2927 | *S. pneumoniae,* serotype 1, encapsulated | CDC |
| TH4848 | *S. pneumoniae,* serotype 9N, encapsulated | SSI |
| TH4849 | *S. pneumoniae,* serotype 11A, encapsulated | SSI |
| TH4850 | *S. pneumoniae,* serotype 17F, encapsulated | SSI |
| TH4702 | D39s derivative, replacing the *dexB-cps2A* region with JC**^b^**, Δ*dexB-cps2A*::JC, Kan^R^ | (12) |
| TH4525 | TH4702 derivative, removing the entire *cps* promoter*,* Sm^R^ | (12) |
| TH4766 | TH4702 derivative, containing type-4 *cps* promoter, Sm^R^ | (12) |
| TH4767 | TH4702 derivative, containing type-6B *cps* promoter, Sm^R^ | This study |
| TH4768 | TH4702 derivative, containing type-14 *cps* promoter, Sm^R^ | This study |
| TH4769 | TH4702 derivative, containing type-18C *cps* promoter, Sm^R^ | This study |
| TH4770 | TH4702 derivative, containing type-19F *cps* promoter, Sm^R^ | This study |
| TH4772 | TH4702 derivative, containing type-6B *cps* promoter, Sm^R^ | This study |
| TH4773 | TH4702 derivative, containing type-6B *cps* promoter, Sm^R^ | This study |
| TH4774 | TH4702 derivative, containing type-6B *cps* promoter, Sm^R^ | This study |
| TH5121 | TH4702 derivative, containing type-8 *cps* promoter, Sm^R^ | This study |
| TH5123 | TH4702 derivative, containing type-3 *cps* promoter, Sm^R^ | This study |
| TH5124 | TH4702 derivative, containing type-19A *cps* promoter, Sm^R^ | This study |
| TH5207 | TH4702 derivative, containing type-22F *cps* promoter, Sm^R^ | This study |
| TH5217 | TH4702 derivative, containing type-11A *cps* promoter, Sm^R^ | This study |
| TH5222 | TH4702 derivative, containing type-1 *cps* promoter, Sm^R^ | This study |
| TH7052 | TH4702 derivative, containing type-6B *cps* promoter, Sm^R^ | This study |
| TH7180 | ST858s derivative, replacing the *dexB-cps6BA* region with JC, Δ*dexB-cps6BA*::JC, Kan^R^ | This study |
| TH7181 | ST858 derivative, *rpsL1,* ST858s, Sm^R^ | This study |
| TH7184 | TH7180 derivative, containing type-6B *cps* promoter, Sm^R^ | This study |
| TH7236 | TH7180 derivative, removing the entire *cps* promoter*,* Sm^R^ | This study |
| TH7239 | TH7180 derivative, containing type-6B *cps* promoter, Sm^R^ | This study |
| TH7242 | TH7180 derivative, containing type-6B *cps* promoter, Sm^R^ | This study |
| TH7331 | TH7180 derivative, containing type-6B *cps* promoter, Sm^R^ | This study |

a. Antibiotic resistance markers: Kan^R^, kanamycin (400 μg/ml); Sm^R^, streptomycin (150 μg/ml)

b. JC, Janus cassette.

c. SSI, Statens Serum Institut

**Table S2. Plasmids used in this study**

| **Plasmids** | **Description^a^** | **Reference or source** |
| --- | --- | --- |
| pTH3932 | pIB166 derivative, containing type-2 *cps* promoter and *luc* | (12) |
| pTH3936 | pTH3932 derivative, lacking the *cps* promoter and endogenous promoter upstream of the multiple cloning site | (12) |
| pTH3937 | pTH3932 derivative, containing type-2 *cps* promoter | (12) |
| pTH3944 | pTH3932 derivative, containing type-4 *cps* promoter | This study |
| pTH3946 | pTH3932 derivative, containing type-19A *cps* promoter | This study |
| pTH3948 | pTH3932 derivative, containing type-9V *cps* promoter | This study |
| pTH3953 | pTH3932 derivative, containing type-23F *cps* promoter | This study |
| pTH3954 | pTH3932 derivative, containing type-19F *cps* promoter | This study |
| pTH3955 | pTH3932 derivative, containing type-7F *cps* promoter | This study |
| pTH4159 | pTH3932 derivative, containing type-1 *cps* promoter | This study |
| pTH4239 | pTH3932 derivative, containing type-18C *cps* promoter | This study |
| pTH4706 | pTH3932 derivative, containing type-33F *cps* promoter | This study |
| pTH4712 | pTH3932 derivative, containing type-8 *cps* promoter | This study |
| pTH4714 | pTH3932 derivative, containing type-15B *cps* promoter | This study |
| pTH4718 | pTH3932 derivative, containing type-10A *cps* promoter | This study |
| pTH4719 | pTH3932 derivative, containing type-20 *cps* promoter | This study |
| pTH4721 | pTH3932 derivative, containing type-6B *cps* promoter | This study |
| pTH4723 | pTH3932 derivative, containing type-12F *cps* promoter | This study |
| pTH4724 | pTH3932 derivative, containing type-14 *cps* promoter | This study |
| pTH4728 | pTH3932 derivative, containing type-22F *cps* promoter | This study |
| pTH4856 | pTH3932 derivative, containing type-3 *cps* promoter | This study |
| pTH4857 | pTH3932 derivative, containing type-9N *cps* promoter | This study |
| pTH4858 | pTH3932 derivative, containing type-11A *cps* promoter | This study |
| pTH4859 | pTH3932 derivative, containing type-17F *cps* promoter | This study |

a. All of the plasmids contained the chloramphenicol acetyltransferase (CAT) gene conferring resistance to chloramphenicol in *S. pneumoniae* (4 μg/ml) and *E. coli* (20 μg/ml)*.*

**Table S3**. **The primers used in this study**

| **Primer ID** | **Sequence (5’-3’)** |
| --- | --- |
| Pr6213 | GAGACCCGGGCATTGAACATCTTACGATTATATCACT |
| Pr6269 | GAGAGGGCCCCAAATCTGTCTTGATTGAAAACA |
| Pr6270 | GAGACCCGGGCCTTGAACATCATACGATTATATCACTTT |
| Pr6271 | GAGACCCGGGCCTTGAACATTGTACGATTATATCACTTT |
| Pr6273 | GAGACCCGGGCATTCAATATCATACGATTATATCACTTT |
| Pr6274 | GAGACCCGGGCCTTGAACATCATACAATTATATCACTTT |
| Pr7321 | GAGACCCGGGCATTGAACACCATACGATTGTAT |
| Pr7322 | GAGACCCGGGCATTGAACATCTTATAATTGTAT |
| Pr7323 | GAGACCCGGGCATTGAACATTACACGATTATATCAC |
| Pr7324 | GAGACCCGGGCATTGAACATCATACGATTATATC |
| Pr7325 | GAGACCCGGGCATTGAACATCATACGATTATATC |
| Pr7339 | TTCCTGACGAGAAGGTAGTCAATAA |
| Pr7340 | TTATAGTAATTCCACACAGAAAGCATCC |
| Pr7343 | TATAGGTGTTAATCATGAGTAGACGTTTT |
| Pr7344 | GTCTAGATGGACATTCCCTACTGGG |
| Pr7350 | GAGACCCGGGCCTTGAACATCGTACAATTATAT |
| Pr7524 | CTTAGTTCCATGGGATGCTTTCTGTGTGGAATTACTATAA |
| Pr7525 | GATTTTTTAAAACGTCTACTCATGATTAACACCTATA |
| Pr7622 | GAGACCCGGGCATTGAACATCATACAATTATATC |
| Pr7662 | TCTGTGTGGAATTACTATAAGTAAAGATTTTTTCAAAAGA |
| Pr7705 | CGCTCAGTGTCGCTGTTTTA |
| Pr7706 | TCTCCCCTGCAATCAAACTC |
| Pr7707 | TGATATCATGGGTGCATTGG |
| Pr7708 | TCCACCCTGCATGGTATTTT |
| Pr7709 | CGACCATCTGGACCAACTTT |
| Pr7710 | CTAGCCAAAGAAGCGACTGC |
| Pr8183 | CAACTAACCCTGCTTCTTGCAACTGACC |

**Table S4**. **The data from cps luciferase, qRT-PCR, immunoblotting, adherence assay, anti-phagocytosis and murine bacteremia model**

| **D39 background** | **cps2A** | **cps2E** | **Encapsulation** | **Adherence/%** | **Phagocytosis/%** | **Virulence/CI** | **luciferase/%** |
| --- | --- | --- | --- | --- | --- | --- | --- |
| 2 | 1.00 | 1.00 | 1.00 | 100 | 100 | 0.51 | 100 |
| Vector/Null | 2.76E-04 | 2.10E-05 | 6.00E-04 | 6077.8 | 117.53 | 9.82E-10 | 6.74 |
| 1 | 1.8 | 1.29 | 1.10 | 88.59 | 84.78 | 0.52 | 21.01 |
| 3 | 2.76 | 2.54 | 1.71 | 85.3 | 56.82 | 0.0018 | 758.41 |
| 4 | 1.25 | 1.03 | 1.13 | 141.38 | 102.19 | 0.49 | 125.03 |
| 6B | 1.31 | 0.95 | 0.32 | 114.79 | 112.25 | 0.1807 | 45.95 |
| 7F | - | - | - | - | - | - | 48.27 |
| 8 | 0.56 | 0.42 | 0.20 | 141.23 | 72.48 | 0.133 | 41.26 |
| 9N | - | - | - | - | - | - | 120.51 |
| 9V | - | - | - | - | - | - | 38.62 |
| 10A | - | - | - | - | - | - | 169.24 |
| 11A | 1.31 | 1.02 | 0.23 | 111.48 | 116.39 | 0.111 | 101.49 |
| 12F | - | - | - | - | - | - | 55.33 |
| 14 | 2.18 | 1.45 | 0.55 | 509.00 | 85.14 | 0.269 | 81.73 |
| 15B | - | - | - | - | - | - | 59.56 |
| 17F | - | - | - | - | - | - | 110.22 |
| 18C | 1.02 | 1.23 | 1.50 | 127.34 | 75.95 | 2.50 | 176.91 |
| 19A | 1.92 | 1.59 | 0.83 | 130.37 | 99.95 | 0.646 | 43.52 |
| 19F | 0.62 | 0.67 | 0.45 | 463.71 | 127.21 | 0.0856 | 80.19 |
| 20 | - | - | - | - | - | - | 84.92 |
| 22F | 0.89 | 1.05 | 0.33 | 155.00 | 84.86 | 0.0469 | 33.89 |
| 23F | 1.57 | 1.76 | 0.23 | 156.15 | 94.29 | 0.0456 | 40.43 |
| 33F | 0.98 | 0.89 | 0.13 | 80.52 | 97.98 | 8.37E-05 | 34.24 |
|  | | | | | | | |
| **D39 background** | **cps2A** | **cps2E** | **Encapsulation** | **Adherence/%** | **Phagocytosis/%** | **Virulence/CI** | **luciferase/%** |
| 6B-1 | 1.00 | 1.00 | 1.00 | 101.83 | 100.00 | 0.1384 | - |
| Null | 2.85E-04 | 1.11E-03 | 0.026 | 6152.06 | 112.85 | 9.82E-07 | - |
| 6B-2 | 0.96 | 1.06 | 1.67 | 57.14 | 168.75 | 3.968 | - |
| 6B-3 | 1.41 | 1.55 | 1.41 | 69.92 | 172.77 | 0.13339 | - |
| 6B-4 | 1.15 | 1.32 | 1.9 | 59.12 | 104.92 | 7.757 | - |
| 6B-5 | 1.07 | 1.17 | 1.41 | 49.08 | 95.05 | 0.334 | - |
|  | | | | | | | |
| **6B background** | **cps6BA** | **cps6BE** | **Encapsulation** | **Adherence/%** | **Phagocytosis/%** | **Virulence/CI** | **luciferase/%** |
| 6B-1 | 1.08 | 1.00 | 1.00 | 100.00 | 100.00 | 0.202 | - |
| Null | 0.0053 | 0.001 | 0.099 | 1210.69 | 252.31 | 6.87E-04 | - |
| 6B-2 | 1.1 | 1.31 | 1.04 | 121.41 | 108.3 | 0.1226 | - |
| 6B-3 | 1.13 | 1.05 | 0.375 | 43.92 | 274.16 | 0.012 | - |
| 6B-4 | 1.17 | 1.21 | 0.26 | 472.07 | 135.68 | 0.0382 | - |
| 6B-5 | 1.00 | 1.35 | 0.291 | 64.75 | 79.03 | 0.0616 | - |
